# Supplementary material for: Multi-omics analyses reveal that the gut microbiome and its metabolites promote milk fat synthesis in Zhongdian yak cows
Source: PeerJ. 2022 Dec 2;10:e14444. doi: 10.7717/peerj.14444 (PMC9744170; doi:10.7717/peerj.14444)
Supplement: Supplemental Information 13 [file peerj-10-14444-s013.zip › Web_Report/HTML/meta_hmdb_anno.xls.html]

meta\_hmdb\_anno.xls


## meta\_hmdb\_anno.xls

| #ID | Name | HMDB\_ID | kingdom | super\_class | class | sub\_class | pubchem\_compound\_id | cas\_registry\_number |
| --- | --- | --- | --- | --- | --- | --- | --- | --- |
| pos\_1 | D-erythro-Dihydrosphingosine | HMDB0000269 | Organic compounds | Organic nitrogen compounds | Organonitrogen compounds | Amines | 91486 | 764-22-7 |
| pos\_1001 | Hexylamine | HMDB0032323 | Organic compounds | Organic nitrogen compounds | Organonitrogen compounds | Amines | 8102 | 111-26-2 |
| pos\_1004 | DG(20:4(5Z,8Z,11Z,14Z)/22:5(4Z,7Z,10Z,13Z,16Z)/0:0) | HMDB0007525 | Organic compounds | Lipids and lipid-like molecules | Glycerolipids | Diradylglycerols | 53478305 | \_ |
| pos\_1005 | Bovinic acid | HMDB0003797 | Organic compounds | Lipids and lipid-like molecules | Fatty Acyls | Lineolic acids and derivatives | 5280644 | 2540-56-9 |
| pos\_1007 | PE(14:0/22:4(7Z,10Z,13Z,16Z)) | HMDB0008844 | Organic compounds | Lipids and lipid-like molecules | Glycerophospholipids | Glycerophosphoethanolamines | 52924126 | \_ |
| pos\_1009 | Desvenlafaxine | HMDB0015646 | Organic compounds | Organic oxygen compounds | Organooxygen compounds | Alcohols and polyols | 125017 | 93413-62-8 |
| pos\_1017 | PE(15:0/18:4(6Z,9Z,12Z,15Z)) | HMDB0008898 | Organic compounds | Lipids and lipid-like molecules | Glycerophospholipids | Glycerophosphoethanolamines | 52924165 | \_ |
| pos\_1018 | PC(22:2(13Z,16Z)/22:5(7Z,10Z,13Z,16Z,19Z)) | HMDB0008615 | Organic compounds | Lipids and lipid-like molecules | Glycerophospholipids | Glycerophosphocholines | 53479237 | \_ |
| pos\_1025 | Deoxyadenosine | HMDB0000101 | Organic compounds | Nucleosides, nucleotides, and analogues | Purine nucleosides | Purine 2'-deoxyribonucleosides | 13730 | 958-09-8 |
| pos\_1026 | DG(18:3(6Z,9Z,12Z)/20:3(5Z,8Z,11Z)/0:0) | HMDB0007284 | Organic compounds | Lipids and lipid-like molecules | Glycerolipids | Diradylglycerols | 53478126 | \_ |
| pos\_1027 | 2-Oxoarginine | HMDB0004225 | Organic compounds | Organic acids and derivatives | Keto acids and derivatives | Short-chain keto acids and derivatives | 558 | 3715-10-4 |
| pos\_1036 | Amdinocillin | HMDB0015294 | Organic compounds | Organic acids and derivatives | Carboxylic acids and derivatives | Amino acids, peptides, and analogues | 36273 | 32887-01-7 |
| pos\_1041 | TG(22:0/14:1(9Z)/20:5(5Z,8Z,11Z,14Z,17Z)) | HMDB0046452 | Organic compounds | Lipids and lipid-like molecules | Glycerolipids | Triradylcglycerols | 131757375 | \_ |
| pos\_1046 | Lucyoside R | HMDB0032836 | Organic compounds | Lipids and lipid-like molecules | Prenol lipids | Terpene glycosides | 73745597 | 206270-86-2 |
| pos\_1050 | Brassinolide | HMDB0041130 | Organic compounds | Lipids and lipid-like molecules | Steroids and steroid derivatives | Steroid lactones | 3239 | 78821-43-9 |
| pos\_1062 | Annosquamosin B | HMDB0031379 | Organic compounds | Lipids and lipid-like molecules | Prenol lipids | Diterpenoids | 51136563 | 177742-56-2 |
| pos\_1067 | PE(22:0/24:1(15Z)) | HMDB0009509 | Organic compounds | Lipids and lipid-like molecules | Glycerophospholipids | Glycerophosphoethanolamines | 9546991 | \_ |
| pos\_1085 | Arginyl-Proline | HMDB0028717 | Organic compounds | Organic acids and derivatives | Carboxylic acids and derivatives | Amino acids, peptides, and analogues | 151003 | 2418-69-1 |
| pos\_1092 | Octreotide | HMDB0014262 | Organic compounds | Organic acids and derivatives | Carboxylic acids and derivatives | Amino acids, peptides, and analogues | 6400441 | 83150-76-9 |
| pos\_1097 | DG(15:0/18:4(6Z,9Z,12Z,15Z)/0:0) | HMDB0007077 | Organic compounds | Lipids and lipid-like molecules | Fatty Acyls | Lineolic acids and derivatives | 53478006 | \_ |
| pos\_1100 | 3a,7a-Dihydroxycoprostanic acid | HMDB0000359 | Organic compounds | Lipids and lipid-like molecules | Steroids and steroid derivatives | Bile acids, alcohols and derivatives | 5284239 | 17974-66-2 |
| pos\_111 | PE(16:0/0:0) | HMDB0011503 | Organic compounds | Lipids and lipid-like molecules | Glycerophospholipids | Glycerophosphoethanolamines | 9547069 | 53862-35-4 |
| pos\_1115 | PG(16:1(9Z)/18:1(11Z)) | HMDB0010588 | Organic compounds | Lipids and lipid-like molecules | Glycerophospholipids | Glycerophosphoglycerols | 53480606 | \_ |
| pos\_112 | Hyacinthin | HMDB0006236 | Organic compounds | Benzenoids | Benzene and substituted derivatives | Phenylacetaldehydes | 998 | 122-78-1 |
| pos\_1125 | PS(18:1(9Z)/18:2(9Z,12Z)) | HMDB0012391 | Organic compounds | Lipids and lipid-like molecules | Glycerophospholipids | Glycerophosphoserines | 52926065 | \_ |
| pos\_1129 | Octanoylglucuronide | HMDB0010347 | Organic compounds | Organic oxygen compounds | Organooxygen compounds | Carbohydrates and carbohydrate conjugates | 127448 | 99910-55-1 |
| pos\_1131 | DG(22:6(4Z,7Z,10Z,13Z,16Z,19Z)/18:0/0:0) | HMDB0007767 | Organic compounds | Lipids and lipid-like molecules | Glycerolipids | Diradylglycerols | 53478518 | \_ |
| pos\_1136 | Uralenolide | HMDB0038797 | Organic compounds | Lipids and lipid-like molecules | Steroids and steroid derivatives | \_ | 131752468 | 111150-27-7 |
| pos\_1138 | Epitheaflagallin 3-O-gallate | HMDB0033288 | Organic compounds | Benzenoids | Benzene and substituted derivatives | Benzoic acids and derivatives | 14520971 | 102067-92-5 |
| pos\_1143 | PE(15:0/20:2(11Z,14Z)) | HMDB0008901 | Organic compounds | Lipids and lipid-like molecules | Glycerophospholipids | Glycerophosphoethanolamines | 52924168 | \_ |
| pos\_1145 | PC(15:0/22:5(4Z,7Z,10Z,13Z,16Z)) | HMDB0007956 | Organic compounds | Lipids and lipid-like molecules | Glycerophospholipids | Glycerophosphocholines | 53478653 | \_ |
| pos\_1147 | DG(14:1(9Z)/15:0/0:0) | HMDB0007039 | Organic compounds | Lipids and lipid-like molecules | Glycerolipids | Diradylglycerols | 53477974 | \_ |
| pos\_1153 | 4-Hydroxydebrisoquine | HMDB0006468 | Organic compounds | Organoheterocyclic compounds | Tetrahydroisoquinolines | \_ | 107669 | 59333-79-8 |
| pos\_1159 | DG(18:1n7/0:0/18:2n6) | HMDB0056183 | Organic compounds | Lipids and lipid-like molecules | Glycerolipids | Diradylglycerols | 131801892 | \_ |
| pos\_1160 | Threoninyl-Glycine | HMDB0029061 | Organic compounds | Organic acids and derivatives | Carboxylic acids and derivatives | Amino acids, peptides, and analogues | 7010575 | 686-44-2 |
| pos\_1161 | PS(18:0/18:3(9Z,12Z,15Z)) | HMDB0012381 | Organic compounds | Lipids and lipid-like molecules | Glycerophospholipids | Glycerophosphoserines | 52925426 | \_ |
| pos\_1165 | Estradiol | HMDB0000151 | Organic compounds | Lipids and lipid-like molecules | Steroids and steroid derivatives | Estrane steroids | 5757 | 50-28-2 |
| pos\_1166 | Neogrifolin | HMDB0030053 | Organic compounds | Lipids and lipid-like molecules | Prenol lipids | Sesquiterpenoids | 9874426 | 23665-96-5 |
| pos\_1169 | Testosterone | HMDB0002833 | Organic compounds | Lipids and lipid-like molecules | Steroids and steroid derivatives | Sulfated steroids | 119207 | 651-45-6 |
| pos\_117 | 4'-METHOXYCHALCONE | HMDB0032585 | Organic compounds | Phenylpropanoids and polyketides | Linear 1,3-diarylpropanoids | Chalcones and dihydrochalcones | 641818 | 22966-19-4 |
| pos\_118 | Oxindole | HMDB0061918 | Organic compounds | Organoheterocyclic compounds | Indoles and derivatives | Indolines | 321710 | 59-48-3 |
| pos\_1180 | Ustiloxin B | HMDB0041373 | Organic compounds | Lipids and lipid-like molecules | Fatty Acyls | Fatty alcohols | 85067000 | 151841-41-7 |
| pos\_1193 | Glabrene | HMDB0029530 | Organic compounds | Phenylpropanoids and polyketides | Isoflavonoids | Pyranoisoflavonoids | 480774 | 60008-03-9 |
| pos\_1195 | 3-Succinoylpyridine | HMDB0000992 | Organic compounds | Organic acids and derivatives | Keto acids and derivatives | Gamma-keto acids and derivatives | 437 | 4192-31-8 |
| pos\_1201 | TG(8:0/8:0/i-17:0) | HMDB0071439 | Organic compounds | Lipids and lipid-like molecules | Glycerolipids | Triradylcglycerols | 131778244 | \_ |
| pos\_1204 | Androsterone | HMDB0000031 | Organic compounds | Lipids and lipid-like molecules | Steroids and steroid derivatives | Androstane steroids | 5879 | 53-41-8 |
| pos\_1207 | PC(18:4(6Z,9Z,12Z,15Z)/22:6(4Z,7Z,10Z,13Z,16Z,19Z)) | HMDB0008255 | Organic compounds | Lipids and lipid-like molecules | Glycerophospholipids | Glycerophosphocholines | 52922939 | \_ |
| pos\_1210 | Alloxanthin | HMDB0036874 | Organic compounds | Lipids and lipid-like molecules | Prenol lipids | Diterpenoids | 12305997 | 28380-31-6 |
| pos\_1215 | O-Ureidohomoserine | HMDB0012271 | Organic compounds | Organic acids and derivatives | Carboxylic acids and derivatives | Amino acids, peptides, and analogues | 21126175 | 51767-67-0 |
| pos\_1230 | gamma-Chaconine | HMDB0033678 | Organic compounds | Lipids and lipid-like molecules | Steroids and steroid derivatives | Steroidal glycosides | 12302826 | 511-36-4 |
| pos\_1237 | Steviobioside | HMDB0036707 | Organic compounds | Lipids and lipid-like molecules | Prenol lipids | Terpene glycosides | 16401639 | 41093-60-1 |
| pos\_1241 | Acarbose | HMDB0014429 | Organic compounds | Organic oxygen compounds | Organooxygen compounds | Carbohydrates and carbohydrate conjugates | 441184 | 56180-94-0 |
| pos\_1247 | PE(18:3(6Z,9Z,12Z)/22:5(4Z,7Z,10Z,13Z,16Z)) | HMDB0009142 | Organic compounds | Lipids and lipid-like molecules | Glycerophospholipids | Glycerophosphoethanolamines | 53479677 | \_ |
| pos\_1248 | Cerebroside B | HMDB0035990 | Organic compounds | Lipids and lipid-like molecules | Sphingolipids | Glycosphingolipids | 10010147 | 88642-46-0 |
| pos\_1250 | Valyl-Threonine | HMDB0029137 | Organic compounds | Organic acids and derivatives | Carboxylic acids and derivatives | Amino acids, peptides, and analogues | \_ | 72636-02-3 |
| pos\_1251 | Ganoderic acid H | HMDB0035987 | Organic compounds | Lipids and lipid-like molecules | Prenol lipids | Triterpenoids | 73657194 | 98665-19-1 |
| pos\_1252 | Docosahexaenoic acid | HMDB0002183 | Organic compounds | Lipids and lipid-like molecules | Fatty Acyls | Fatty acids and conjugates | 445580 | 6217-54-5 |
| pos\_1254 | Dihydrocapsiate | HMDB0034781 | Organic compounds | Benzenoids | Phenols | Methoxyphenols | 9873754 | \_ |
| pos\_1257 | PE(20:1(11Z)/22:6(4Z,7Z,10Z,13Z,16Z,19Z)) | HMDB0009276 | Organic compounds | Lipids and lipid-like molecules | Glycerophospholipids | Glycerophosphoethanolamines | 52924571 | \_ |
| pos\_1260 | Vitamin A2 | HMDB0013117 | Organic compounds | Lipids and lipid-like molecules | Prenol lipids | Retinoids | 6436043 | 79-80-1 |
| pos\_1264 | 1-Methylhistidine | HMDB0000001 | Organic compounds | Organic acids and derivatives | Carboxylic acids and derivatives | Amino acids, peptides, and analogues | 92105 | 332-80-9 |
| pos\_1267 | L-Histidine | HMDB0000177 | Organic compounds | Organic acids and derivatives | Carboxylic acids and derivatives | Amino acids, peptides, and analogues | 6274 | 71-00-1 |
| pos\_1270 | PC(14:1(9Z)/22:5(4Z,7Z,10Z,13Z,16Z)) | HMDB0007923 | Organic compounds | Lipids and lipid-like molecules | Glycerophospholipids | Glycerophosphocholines | 53478631 | \_ |
| pos\_1271 | DG(20:0/0:0/20:2n6) | HMDB0056075 | Organic compounds | Lipids and lipid-like molecules | Fatty Acyls | Eicosanoids | 131801789 | \_ |
| pos\_1275 | L-Histidinol | HMDB0003431 | Organic compounds | Organic nitrogen compounds | Organonitrogen compounds | Amines | 165271 | 4836-52-6 |
| pos\_1276 | PE(16:0/22:5(4Z,7Z,10Z,13Z,16Z)) | HMDB0008944 | Organic compounds | Lipids and lipid-like molecules | Glycerophospholipids | Glycerophosphoethanolamines | 53479589 | \_ |
| pos\_1282 | Dolichyl diphosphate | HMDB0001513 | Organic compounds | Lipids and lipid-like molecules | Prenol lipids | Polyprenols | 24892721 | 37247-98-6 |
| pos\_1285 | 1-Cyano-2-hydroxy-3-butene | HMDB0031339 | Organic compounds | Organic oxygen compounds | Organooxygen compounds | Alcohols and polyols | 91586 | 7451-85-6 |
| pos\_1286 | L-Urobilinogen | HMDB0004157 | Organic compounds | Organoheterocyclic compounds | Tetrapyrroles and derivatives | Bilirubins | 9548718 | 17095-63-5 |
| pos\_1289 | Licorisoflavan A | HMDB0034184 | Organic compounds | Phenylpropanoids and polyketides | Isoflavonoids | O-methylated isoflavonoids | 5319704 | 129314-37-0 |
| pos\_1295 | PE(20:3(8Z,11Z,14Z)/22:0) | HMDB0009369 | Organic compounds | Lipids and lipid-like molecules | Glycerophospholipids | Glycerophosphoethanolamines | 52924629 | \_ |
| pos\_1301 | PS(14:1(9Z)/18:2(9Z,12Z)) | HMDB0012347 | Organic compounds | Lipids and lipid-like molecules | Glycerophospholipids | Glycerophosphoserines | 52925231 | \_ |
| pos\_1307 | DG(14:1(9Z)/20:0/0:0) | HMDB0007049 | Organic compounds | Lipids and lipid-like molecules | Glycerolipids | Diradylglycerols | 53477984 | \_ |
| pos\_1313 | LysoPE(0:0/20:3(11Z,14Z,17Z)) | HMDB0011484 | Organic compounds | Lipids and lipid-like molecules | Glycerophospholipids | Glycerophosphoethanolamines | 53480933 | \_ |
| pos\_1315 | Isopropylmaleate | HMDB0012241 | Organic compounds | Lipids and lipid-like molecules | Fatty Acyls | Fatty acids and conjugates | 5280533 | 44976-69-4 |
| pos\_1318 | Deoxyloganic acid | HMDB0037028 | Organic compounds | Lipids and lipid-like molecules | Prenol lipids | Terpene glycosides | 4490284 | 92842-56-3 |
| pos\_1323 | Guanidinosuccinic acid | HMDB0003157 | Organic compounds | Organic acids and derivatives | Carboxylic acids and derivatives | Amino acids, peptides, and analogues | 439918 | 6133-30-8 |
| pos\_1329 | Indolylacryloylglycine | HMDB0006005 | Organic compounds | Organic acids and derivatives | Carboxylic acids and derivatives | Amino acids, peptides, and analogues | 5370648 | 3475-68-1 |
| pos\_1339 | PE(18:3(6Z,9Z,12Z)/20:5(5Z,8Z,11Z,14Z,17Z)) | HMDB0009137 | Organic compounds | Lipids and lipid-like molecules | Glycerophospholipids | Glycerophosphoethanolamines | 52924404 | \_ |
| pos\_1350 | Nandrolone phenpropionate | HMDB0002725 | Organic compounds | Lipids and lipid-like molecules | Steroids and steroid derivatives | Estrane steroids | 9904 | 434-22-0 |
| pos\_1366 | TG(14:1(9Z)/18:4(6Z,9Z,12Z,15Z)/22:6(4Z,7Z,10Z,13Z,16Z,19Z)) | HMDB0048297 | Organic compounds | Lipids and lipid-like molecules | Glycerolipids | Triradylcglycerols | \_ | \_ |
| pos\_1369 | Alanyltryptophan | HMDB0013209 | Organic compounds | Organic acids and derivatives | Carboxylic acids and derivatives | Amino acids, peptides, and analogues | 85362 | 16305-75-2 |
| pos\_1388 | 2,3-Dehydrosilybin | HMDB0040513 | Organic compounds | Lignans, neolignans and related compounds | Flavonolignans | \_ | 5467200 | 25166-14-7 |
| pos\_1392 | 6-Gingesulfonic acid | HMDB0038999 | Organic compounds | Benzenoids | Phenols | Methoxyphenols | 126890 | 145937-21-9 |
| pos\_1394 | Nandrolone decanoate | HMDB0002725 | Organic compounds | Lipids and lipid-like molecules | Steroids and steroid derivatives | Estrane steroids | 9904 | 434-22-0 |
| pos\_1396 | Myristoylglycine | HMDB0013250 | Organic compounds | Organic acids and derivatives | Carboxylic acids and derivatives | Amino acids, peptides, and analogues | 72348 | \_ |
| pos\_1398 | Gemfibrozil | HMDB0015371 | Organic compounds | Benzenoids | Phenol ethers | \_ | 3463 | 25812-30-0 |
| pos\_14 | Decanoic acid | HMDB0000513 | Organic compounds | Lipids and lipid-like molecules | Fatty Acyls | Fatty acids and conjugates | 2969 | 59906-14-8 |
| pos\_1416 | Ustiloxin A | HMDB0039640 | Organic compounds | Organic acids and derivatives | Carboxylic acids and derivatives | Amino acids, peptides, and analogues | 4480102 | 141044-51-1 |
| pos\_1419 | Lysyl-Valine | HMDB0028964 | Organic compounds | Organic acids and derivatives | Carboxylic acids and derivatives | Amino acids, peptides, and analogues | 6992341 | 20556-11-0 |
| pos\_1421 | DG(22:1(13Z)/22:6(4Z,7Z,10Z,13Z,16Z,19Z)/0:0) | HMDB0007643 | Organic compounds | Lipids and lipid-like molecules | Glycerolipids | Diradylglycerols | 9543962 | \_ |
| pos\_1423 | PI(16:0/20:2(11Z,14Z)) | HMDB0009786 | Organic compounds | Lipids and lipid-like molecules | Glycerophospholipids | Glycerophosphoinositols | \_ | \_ |
| pos\_1425 | Hydroxyhomodestruxin B | HMDB0039897 | Organic compounds | Organic acids and derivatives | Peptidomimetics | Depsipeptides | 85262217 | \_ |
| pos\_1426 | DG(20:5(5Z,8Z,11Z,14Z,17Z)/22:0/0:0) | HMDB0007579 | Organic compounds | Lipids and lipid-like molecules | Glycerolipids | Diradylglycerols | 9543909 | \_ |
| pos\_1427 | Erythrohydrobupropion | HMDB0012227 | Organic compounds | Benzenoids | Benzene and substituted derivatives | Phenylpropanes | 445 | \_ |
| pos\_1428 | Phenylalanyl-Valine | HMDB0029008 | Organic compounds | Organic acids and derivatives | Carboxylic acids and derivatives | Amino acids, peptides, and analogues | 7359121 | 3918-90-9 |
| pos\_1429 | 5-Taurinomethyl-2-thiouridine | HMDB0011610 | Organic compounds | Nucleosides, nucleotides, and analogues | Pyrimidine nucleosides | \_ | 53481004 | 497258-54-5 |
| pos\_1430 | PC(20:4(5Z,8Z,11Z,14Z)/18:0) | HMDB0008431 | Organic compounds | Lipids and lipid-like molecules | Glycerophospholipids | Glycerophosphocholines | 52923291 | \_ |
| pos\_1431 | Niazimicin A | HMDB0031944 | Organic compounds | Organic oxygen compounds | Organooxygen compounds | Carbohydrates and carbohydrate conjugates | 10247749 | 147821-49-6 |
| pos\_1438 | Isometheptene | HMDB0015651 | Organic compounds | Organic nitrogen compounds | Organonitrogen compounds | Amines | 22297 | 503-01-5 |
| pos\_1439 | Glutamyl-Phenylalanine | HMDB0029156 | Organic compounds | Organic acids and derivatives | Carboxylic acids and derivatives | Amino acids, peptides, and analogues | 18653118 | 20556-22-3 |
| pos\_144 | Ethyl 3-(methylthio)propanoate | HMDB0040413 | Organic compounds | Organic acids and derivatives | Carboxylic acids and derivatives | Carboxylic acid derivatives | 61592 | 13327-56-5 |
| pos\_1440 | Ethyl glucuronide | HMDB0131286 | Organic compounds | Organic oxygen compounds | Organooxygen compounds | Carbohydrates and carbohydrate conjugates | 21845293 | \_ |
| pos\_1442 | DG(16:0/18:1(11Z)/0:0) | HMDB0007101 | Organic compounds | Lipids and lipid-like molecules | Glycerolipids | Diradylglycerols | 9543972 | \_ |
| pos\_1444 | Vanillic acid-4-O-glucuronide | HMDB0060024 | Organic compounds | Organic oxygen compounds | Organooxygen compounds | Carbohydrates and carbohydrate conjugates | 101579718 | 18191-12-3 |
| pos\_1445 | Koryoginsenoside R1 | HMDB0041351 | Organic compounds | Lipids and lipid-like molecules | Prenol lipids | Triterpenoids | 131753115 | 171674-97-8 |
| pos\_1447 | 3-alpha-Androstanediol glucuronide | HMDB0010339 | Organic compounds | Alkaloids and derivatives | Morphinans | \_ | 24883428 | \_ |
| pos\_1448 | Valyl-Isoleucine | HMDB0029130 | Organic compounds | Organic acids and derivatives | Carboxylic acids and derivatives | Amino acids, peptides, and analogues | 7010531 | 20556-14-3 |
| pos\_1470 | PE(20:5(5Z,8Z,11Z,14Z,17Z)/24:1(15Z)) | HMDB0009476 | Organic compounds | Lipids and lipid-like molecules | Glycerophospholipids | Glycerophosphoethanolamines | 53479838 | \_ |
| pos\_1479 | Alanyl-Proline | HMDB0028695 | Organic compounds | Organic acids and derivatives | Carboxylic acids and derivatives | Amino acids, peptides, and analogues | 83525 | 13485-59-1 |
| pos\_1481 | delta-Methylionone | HMDB0032397 | Organic compounds | Lipids and lipid-like molecules | Prenol lipids | Sesquiterpenoids | 5463913 | 7784-98-7 |
| pos\_149 | cis-Resveratrol | HMDB0041772 | Organic compounds | Phenylpropanoids and polyketides | Stilbenes | \_ | 25113755 | \_ |
| pos\_1492 | TG(10:0/8:0/8:0) | HMDB0072193 | Organic compounds | Lipids and lipid-like molecules | Glycerolipids | Triradylcglycerols | 73416182 | \_ |
| pos\_1493 | TG(8:0/8:0/i-12:0) | HMDB0071469 | Organic compounds | Lipids and lipid-like molecules | Glycerolipids | Triradylcglycerols | 131778274 | \_ |
| pos\_1498 | PC(DiMe(11,5)/MonoMe(11,3)) | HMDB0061404 | Organic compounds | Lipids and lipid-like molecules | Glycerophospholipids | Glycerophosphocholines | \_ | \_ |
| pos\_1500 | Aesculin | HMDB0030820 | Organic compounds | Phenylpropanoids and polyketides | Coumarins and derivatives | Coumarin glycosides | 5281417 | 531-75-9 |
| pos\_1503 | PE(22:2(13Z,16Z)/22:6(4Z,7Z,10Z,13Z,16Z,19Z)) | HMDB0009573 | Organic compounds | Lipids and lipid-like molecules | Glycerophospholipids | Glycerophosphoethanolamines | 52924802 | \_ |
| pos\_1510 | DG(20:5(5Z,8Z,11Z,14Z,17Z)/22:6(4Z,7Z,10Z,13Z,16Z,19Z)/0:0) | HMDB0007585 | Organic compounds | Lipids and lipid-like molecules | Glycerolipids | Diradylglycerols | 9543951 | \_ |
| pos\_1517 | Dethiobiotin | HMDB0003581 | Organic compounds | Lipids and lipid-like molecules | Fatty Acyls | Fatty acids and conjugates | 445027 | 533-48-2 |
| pos\_1518 | L-phenylalanyl-L-proline | HMDB0011177 | Organic compounds | Organic acids and derivatives | Carboxylic acids and derivatives | Amino acids, peptides, and analogues | 7020641 | 7669-65-0 |
| pos\_152 | Biliverdin IX | HMDB0001008 | Organic compounds | Organoheterocyclic compounds | Tetrapyrroles and derivatives | Bilirubins | 5353439 | 114-25-0 |
| pos\_1521 | Matesaponin 3 | HMDB0040964 | Organic compounds | Lipids and lipid-like molecules | Prenol lipids | Terpene glycosides | 73157196 | 164178-28-3 |
| pos\_1522 | DG(14:0/16:1(9Z)/0:0) | HMDB0007012 | Organic compounds | Lipids and lipid-like molecules | Glycerolipids | Diradylglycerols | 53477950 | \_ |
| pos\_1523 | 3-Hydroxyhexadecanoylcarnitine | HMDB0013336 | Organic compounds | Lipids and lipid-like molecules | Fatty Acyls | Fatty acid esters | 126456228 | 195207-76-2 |
| pos\_1525 | Propylene glycol alginate | HMDB0039860 | Organic compounds | Organic oxygen compounds | Organooxygen compounds | Carbohydrates and carbohydrate conjugates | 131752735 | \_ |
| pos\_153 | Ursolic acid | HMDB0002395 | Organic compounds | Lipids and lipid-like molecules | Prenol lipids | Triterpenoids | 64945 | 77-52-1 |
| pos\_154 | Necatorine | HMDB0031330 | Organic compounds | Organohalogen compounds | Alkyl halides | Halomethanes | 5943 | 56-23-5 |
| pos\_1541 | Tryptophanol | HMDB0003447 | Organic compounds | Organoheterocyclic compounds | Indoles and derivatives | Indoles | 10685 | 526-55-6 |
| pos\_1543 | Monomenthyl succinate | HMDB0036143 | Organic compounds | Lipids and lipid-like molecules | Prenol lipids | Monoterpenoids | 10199004 | 188709-97-9 |
| pos\_155 | Alpha-Calacorene | HMDB0036453 | Organic compounds | Lipids and lipid-like molecules | Prenol lipids | Sesquiterpenoids | 528708 | 21391-99-1 |
| pos\_1565 | DG(18:1(11Z)/22:4(7Z,10Z,13Z,16Z)/0:0) | HMDB0007205 | Organic compounds | Lipids and lipid-like molecules | Glycerolipids | Diradylglycerols | 53478080 | \_ |
| pos\_1566 | (-)-alpha-Narcotine | HMDB0033439 | Organic compounds | Alkaloids and derivatives | Phthalide isoquinolines | \_ | 275196 | 128-62-1 |
| pos\_1569 | Propoxyphene | HMDB0001847 | Organic compounds | Organoheterocyclic compounds | Imidazopyrimidines | Purines and purine derivatives | 2519 | 58-08-2 |
| pos\_1570 | Panaxynol | HMDB0039588 | Organic compounds | Lipids and lipid-like molecules | Fatty Acyls | Fatty alcohols | 5353620 | 81203-57-8 |
| pos\_1573 | Nicotinuric acid | HMDB0003269 | Organic compounds | Organic acids and derivatives | Carboxylic acids and derivatives | Amino acids, peptides, and analogues | 68499 | 583-08-4 |
| pos\_1579 | Ginsenoside Rh1 | HMDB0039554 | Organic compounds | Lipids and lipid-like molecules | Prenol lipids | Triterpenoids | 12855917 | 63223-86-9 |
| pos\_1581 | Bicine | HMDB0011727 | Organic compounds | Organic acids and derivatives | Carboxylic acids and derivatives | Amino acids, peptides, and analogues | 8761 | 150-25-4 |
| pos\_1588 | Americanin A | HMDB0030834 | Organic compounds | Organoheterocyclic compounds | Benzodioxanes | Phenylbenzodioxanes | 131751085 | 69506-79-2 |
| pos\_159 | 3-Phenoxypropionic acid | HMDB0002229 | Organic compounds | Benzenoids | Benzene and substituted derivatives | 3-phenoxypropionic acids | 81596 | 7170-38-9 |
| pos\_1593 | PG(16:0/18:2(9Z,12Z)) | HMDB0010575 | Organic compounds | Lipids and lipid-like molecules | Glycerophospholipids | Glycerophosphoglycerols | 52927246 | \_ |
| pos\_16 | Rosmarinic acid | HMDB0003572 | Organic compounds | Phenylpropanoids and polyketides | Cinnamic acids and derivatives | Hydroxycinnamic acids and derivatives | 5281792 | 20283-92-5 |
| pos\_1601 | Prostaglandin E1 | HMDB0001442 | Organic compounds | Lipids and lipid-like molecules | Fatty Acyls | Eicosanoids | 5280723 | 745-65-3 |
| pos\_1604 | PS(20:3(8Z,11Z,14Z)/18:0) | HMDB0012422 | Organic compounds | Lipids and lipid-like molecules | Glycerophospholipids | Glycerophosphoserines | 52925710 | \_ |
| pos\_1608 | 3-Methyl-2-cyclohexen-1-one | HMDB0031541 | Organic compounds | Organic oxygen compounds | Organooxygen compounds | Carbonyl compounds | 14511 | 1193-18-6 |
| pos\_1616 | DG(14:0/20:5(5Z,8Z,11Z,14Z,17Z)/0:0) | HMDB0007027 | Organic compounds | Lipids and lipid-like molecules | Glycerolipids | Diradylglycerols | 53477962 | \_ |
| pos\_1619 | LysoPC(20:0) | HMDB0010390 | Organic compounds | Lipids and lipid-like molecules | Glycerophospholipids | Glycerophosphocholines | 24779473 | 108341-80-6 |
| pos\_1622 | Dimethylamphetamine | HMDB0041880 | Organic compounds | Benzenoids | Benzene and substituted derivatives | Phenethylamines | 20006 | 4075-96-1 |
| pos\_1630 | PE(14:1(9Z)/22:1(13Z)) | HMDB0008875 | Organic compounds | Lipids and lipid-like molecules | Glycerophospholipids | Glycerophosphoethanolamines | 53479566 | \_ |
| pos\_1640 | Curdione | HMDB0034714 | Organic compounds | Lipids and lipid-like molecules | Prenol lipids | Sesquiterpenoids | 5362828 | 13657-68-6 |
| pos\_1650 | Laurotetanine | HMDB0030220 | Organic compounds | Alkaloids and derivatives | Aporphines | \_ | 267400 | 128-76-7 |
| pos\_1652 | (7Z,10Z,13Z,16Z)-docosatetraenoate | HMDB0002226 | Organic compounds | Lipids and lipid-like molecules | Fatty Acyls | Fatty acids and conjugates | 5497181 | 28874-58-0 |
| pos\_1658 | Serratol | HMDB0039827 | Organic compounds | Lipids and lipid-like molecules | Prenol lipids | Diterpenoids | 101618281 | 67814-27-1 |
| pos\_1661 | PGP(16:1(9Z)/18:2(9Z,12Z)) | HMDB0013492 | Organic compounds | Lipids and lipid-like molecules | Glycerophospholipids | Glycerophosphoglycerophosphates | 53481812 | \_ |
| pos\_1662 | Linoleoyl ethanolamide | HMDB0013624 | Organic compounds | Organic nitrogen compounds | Organonitrogen compounds | Amines | 5283449 | \_ |
| pos\_1664 | PC(15:0/15:0) | HMDB0007934 | Organic compounds | Lipids and lipid-like molecules | Glycerophospholipids | Glycerophosphocholines | 24778654 | \_ |
| pos\_1668 | LysoPE(0:0/20:0) | HMDB0011481 | Organic compounds | Lipids and lipid-like molecules | Glycerophospholipids | Glycerophosphoethanolamines | 53480930 | \_ |
| pos\_1674 | (3S)-3,6-Diaminohexanoate | HMDB0012114 | Organic compounds | Organic acids and derivatives | Carboxylic acids and derivatives | Amino acids, peptides, and analogues | 439417 | 4299-56-3 |
| pos\_1675 | 3-Sulfinato-L-alaninate | HMDB0000996 | Organic compounds | Organic acids and derivatives | Carboxylic acids and derivatives | Amino acids, peptides, and analogues | 1549098 | 1115-65-7 |
| pos\_1678 | Retinyl beta-glucuronide | HMDB0010340 | Organic compounds | Lipids and lipid-like molecules | Prenol lipids | Terpene glycosides | 6440956 | \_ |
| pos\_1684 | PC(14:1(9Z)/22:1(13Z)) | HMDB0007920 | Organic compounds | Lipids and lipid-like molecules | Glycerophospholipids | Glycerophosphocholines | 53478629 | \_ |
| pos\_1687 | Safflomin A | HMDB0040677 | Organic compounds | Phenylpropanoids and polyketides | Cinnamic acids and derivatives | Hydroxycinnamic acids and derivatives | \_ | 146087-19-6 |
| pos\_1696 | Alanyl-Glutamine | HMDB0028685 | Organic compounds | Organic acids and derivatives | Carboxylic acids and derivatives | Amino acids, peptides, and analogues | 123935 | 39537-23-0 |
| pos\_1699 | Vaporole | HMDB0001382 | Organic compounds | Organic nitrogen compounds | Organonitrogen compounds | Organic nitroso compounds | 8053 | 110-46-3 |
| pos\_1705 | 11,14,17-Eicosatrienoic acid | HMDB0060039 | Organic compounds | Lipids and lipid-like molecules | Fatty Acyls | Fatty acids and conjugates | 5312529 | 17046-59-2 |
| pos\_1709 | SM(d18:1/18:1(11Z)) | HMDB0012100 | Organic compounds | Lipids and lipid-like molecules | Sphingolipids | Phosphosphingolipids | 53481366 | \_ |
| pos\_1711 | DG(14:0/22:1(13Z)/0:0) | HMDB0007029 | Organic compounds | Lipids and lipid-like molecules | Glycerolipids | Diradylglycerols | 53477964 | \_ |
| pos\_1713 | Eicosapentaenoyl Ethanolamide | HMDB0013649 | Organic compounds | Organic nitrogen compounds | Organonitrogen compounds | Amines | 5283450 | 109001-03-8 |
| pos\_1716 | PE(18:1(11Z)/22:5(4Z,7Z,10Z,13Z,16Z)) | HMDB0009043 | Organic compounds | Lipids and lipid-like molecules | Glycerophospholipids | Glycerophosphoethanolamines | 53479641 | \_ |
| pos\_1717 | 2-Isopropylmalic acid | HMDB0000402 | Organic compounds | Lipids and lipid-like molecules | Fatty Acyls | Fatty acids and conjugates | 5280523 | 49601-06-1 |
| pos\_172 | N-Methyl-2-pyrrolidinone | HMDB0002039 | Organic compounds | Organoheterocyclic compounds | Pyrrolidines | Pyrrolidones | 12025 | 616-45-5 |
| pos\_1720 | N-arachidonoylglycinate | HMDB0005096 | Organic compounds | Organic acids and derivatives | Carboxylic acids and derivatives | Amino acids, peptides, and analogues | 5283389 | 179113-91-8 |
| pos\_1723 | 4-cholesten-7α,12α,24-triol-3-one | HMDB0062399 | Organic compounds | Lipids and lipid-like molecules | Steroids and steroid derivatives | Bile acids, alcohols and derivatives | 24755588 | \_ |
| pos\_1729 | hydroxyitraconazole | HMDB0061035 | Organic compounds | Organoheterocyclic compounds | Diazinanes | Piperazines | 108222 | \_ |
| pos\_173 | 5,7,4'-TRIMETHOXYFLAVONE | HMDB0030842 | Organic compounds | Phenylpropanoids and polyketides | oids | O-methylated flavonoids | 79730 | 5631-70-9 |
| pos\_1737 | PE(20:0/22:5(4Z,7Z,10Z,13Z,16Z)) | HMDB0009241 | Organic compounds | Lipids and lipid-like molecules | Glycerophospholipids | Glycerophosphoethanolamines | 53479711 | \_ |
| pos\_1740 | Thiamine monophosphate | HMDB0002666 | Organic compounds | Organoheterocyclic compounds | Diazines | Pyrimidines and pyrimidine derivatives | 1131 | 495-23-8 |
| pos\_1750 | Bifonazole | HMDB0015583 | Organic compounds | Benzenoids | Benzene and substituted derivatives | Diphenylmethanes | 2378 | 60628-96-8 |
| pos\_1751 | Levonorgestrel | HMDB0014511 | Organic compounds | Lipids and lipid-like molecules | Steroids and steroid derivatives | Estrane steroids | 13109 | 797-63-7 |
| pos\_1752 | Alpha-Tocotrienol | HMDB0006327 | Organic compounds | Lipids and lipid-like molecules | Prenol lipids | Quinone and hydroquinone lipids | 5282347 | 58864-81-6 |
| pos\_1763 | Maysin | HMDB0037419 | Organic compounds | Phenylpropanoids and polyketides | Flavonoids | Flavonoid glycosides | 194340 | 70255-49-1 |
| pos\_1790 | DG(20:3(5Z,8Z,11Z)/22:2(13Z,16Z)/0:0) | HMDB0007465 | Organic compounds | Lipids and lipid-like molecules | Glycerolipids | Diradylglycerols | 53478260 | \_ |
| pos\_1795 | PI(16:0/16:1(9Z)) | HMDB0009779 | Organic compounds | Lipids and lipid-like molecules | Glycerophospholipids | Glycerophosphoinositols | \_ | \_ |
| pos\_1799 | Arjunolic acid | HMDB0034502 | Organic compounds | Lipids and lipid-like molecules | Prenol lipids | Triterpenoids | 5320146 | 465-00-9 |
| pos\_1800 | LysoSM(d18:0) | HMDB0012082 | Organic compounds | Lipids and lipid-like molecules | Sphingolipids | Phosphosphingolipids | 44260122 | \_ |
| pos\_1806 | PC(22:5(4Z,7Z,10Z,13Z,16Z)/24:1(15Z)) | HMDB0008684 | Organic compounds | Lipids and lipid-like molecules | Glycerophospholipids | Glycerophosphocholines | 53479331 | \_ |
| pos\_1819 | PS(14:0/18:3(9Z,12Z,15Z)) | HMDB0012337 | Organic compounds | Lipids and lipid-like molecules | Glycerophospholipids | Glycerophosphoserines | 52926033 | \_ |
| pos\_1831 | Cycloalliin | HMDB0029417 | Organic compounds | Phenylpropanoids and polyketides | Flavonoids | Flavonoid glycosides | 3549960 | 455-41-4 |
| pos\_1832 | TG(20:0/a-13:0/8:0) | HMDB0062962 | Organic compounds | Lipids and lipid-like molecules | Glycerolipids | Triradylcglycerols | 131770637 | \_ |
| pos\_1833 | PC(22:5(4Z,7Z,10Z,13Z,16Z)/22:6(4Z,7Z,10Z,13Z,16Z,19Z)) | HMDB0008682 | Organic compounds | Lipids and lipid-like molecules | Glycerophospholipids | Glycerophosphocholines | 53479327 | \_ |
| pos\_1835 | Calcidiol | HMDB0003550 | Organic compounds | Lipids and lipid-like molecules | Steroids and steroid derivatives | Vitamin D and derivatives | 5283731 | 19356-17-3 |
| pos\_1836 | Achyranthoside C | HMDB0031024 | Organic compounds | Lipids and lipid-like molecules | Prenol lipids | Terpene glycosides | 78172884 | 168111-48-6 |
| pos\_1842 | Sinigrin | HMDB0034070 | Organic compounds | Organic oxygen compounds | Organooxygen compounds | Carbohydrates and carbohydrate conjugates | 6911854 | 534-69-0 |
| pos\_1844 | Vitamin K1 | HMDB0003555 | Organic compounds | Lipids and lipid-like molecules | Prenol lipids | Quinone and hydroquinone lipids | 5284607 | 84-80-0 |
| pos\_1857 | DG(18:4(6Z,9Z,12Z,15Z)/22:2(13Z,16Z)/0:0) | HMDB0007349 | Organic compounds | Lipids and lipid-like molecules | Glycerolipids | Diradylglycerols | 53478178 | \_ |
| pos\_1861 | DG(20:4(5Z,8Z,11Z,14Z)/22:1(13Z)/0:0) | HMDB0007522 | Organic compounds | Lipids and lipid-like molecules | Glycerolipids | Diradylglycerols | 9543910 | \_ |
| pos\_1865 | PC(18:1(9Z)/18:1(9Z)) | HMDB0000593 | Organic compounds | Lipids and lipid-like molecules | Glycerophospholipids | Glycerophosphocholines | 10350317 | 4235-95-4 |
| pos\_1874 | Sulfametopyrazine | HMDB0014802 | Organic compounds | Benzenoids | Benzene and substituted derivatives | Benzenesulfonamides | 9047 | 152-47-6 |
| pos\_1876 | PGP(16:0/18:1(11Z)) | HMDB0013475 | Organic compounds | Lipids and lipid-like molecules | Glycerophospholipids | Glycerophosphoglycerophosphates | 9547136 | \_ |
| pos\_1881 | Cryptochrome | HMDB0029857 | Organic compounds | Lipids and lipid-like molecules | Prenol lipids | Tetraterpenoids | 131750919 | 73745-06-9 |
| pos\_1889 | 14,17-epoxy-15-methyldocosa-14,16-dienoic acid | HMDB0061648 | Organic compounds | Lipids and lipid-like molecules | Fatty Acyls | Fatty acids and conjugates | 71443943 | 57818-42-5 |
| pos\_1891 | PC(18:1(11Z)/22:5(4Z,7Z,10Z,13Z,16Z)) | HMDB0008088 | Organic compounds | Lipids and lipid-like molecules | Glycerophospholipids | Glycerophosphocholines | 53478751 | \_ |
| pos\_1896 | Glycyl-glycine | HMDB0011733 | Organic compounds | Organic acids and derivatives | Carboxylic acids and derivatives | Amino acids, peptides, and analogues | 11163 | 556-50-3 |
| pos\_1898 | PS(18:0/20:0) | HMDB0010164 | Organic compounds | Lipids and lipid-like molecules | Glycerophospholipids | Glycerophosphoserines | 52926069 | \_ |
| pos\_19 | Cellobiose | HMDB0000055 | Organic compounds | Organic oxygen compounds | Organooxygen compounds | Carbohydrates and carbohydrate conjugates | 10712 | 528-50-7 |
| pos\_1902 | Hygromycin B | HMDB0034229 | Organic compounds | Organic oxygen compounds | Organooxygen compounds | Carbohydrates and carbohydrate conjugates | 3659 | 31282-04-9 |
| pos\_1904 | Linoleic acid | HMDB0006270 | Organic compounds | Lipids and lipid-like molecules | Fatty Acyls | Lineolic acids and derivatives | 5282457 | 506-21-8 |
| pos\_1913 | Nummularine B | HMDB0029334 | Organic compounds | Organic acids and derivatives | Carboxylic acids and derivatives | Amino acids, peptides, and analogues | \_ | 53947-96-9 |
| pos\_1921 | Corchoionoside B | HMDB0030975 | Organic compounds | Lipids and lipid-like molecules | Fatty Acyls | Fatty acyl glycosides | 131751110 | 189344-54-5 |
| pos\_1924 | Tyrosyl-Hydroxyproline | HMDB0029106 | Organic compounds | Organic acids and derivatives | Carboxylic acids and derivatives | Amino acids, peptides, and analogues | 69040603 | 936346-34-8 |
| pos\_1926 | PG(16:1(9Z)/18:1(9Z)) | HMDB0010589 | Organic compounds | Lipids and lipid-like molecules | Glycerophospholipids | Glycerophosphoglycerols | 52927167 | \_ |
| pos\_1927 | Glutamyl-Lysine | HMDB0004207 | Organic compounds | Organic acids and derivatives | Carboxylic acids and derivatives | Amino acids, peptides, and analogues | 7015704 | 5891-53-2 |
| pos\_1931 | DG(18:3(6Z,9Z,12Z)/22:5(4Z,7Z,10Z,13Z,16Z)/0:0) | HMDB0007293 | Organic compounds | Lipids and lipid-like molecules | Glycerolipids | Diradylglycerols | 53478135 | \_ |
| pos\_1934 | PC(20:3(5Z,8Z,11Z)/24:0) | HMDB0008388 | Organic compounds | Lipids and lipid-like molecules | Glycerophospholipids | Glycerophosphocholines | 53478997 | \_ |
| pos\_1941 | Argininic acid | HMDB0003148 | Organic compounds | Organic acids and derivatives | Carboxylic acids and derivatives | Amino acids, peptides, and analogues | 160437 | 157-07-3 |
| pos\_1949 | 1-Palmitoylglycerophosphoinositol | HMDB0061695 | Organic compounds | Lipids and lipid-like molecules | Glycerophospholipids | Glycerophosphoinositols | \_ | 1425501-12-7 |
| pos\_1963 | PE(14:0/18:3(6Z,9Z,12Z)) | HMDB0008830 | Organic compounds | Lipids and lipid-like molecules | Glycerophospholipids | Glycerophosphoethanolamines | 52924117 | \_ |
| pos\_1965 | Nephritogenoside | HMDB0041948 | Organic compounds | Lipids and lipid-like molecules | Fatty Acyls | Fatty acyl glycosides | 121932 | 81276-22-4 |
| pos\_1966 | TG(22:4(7Z,10Z,13Z,16Z)/22:4(7Z,10Z,13Z,16Z)/22:6(4Z,7Z,10Z,13Z,16Z,19Z)) | HMDB0054836 | Organic compounds | Lipids and lipid-like molecules | Glycerolipids | Triradylcglycerols | 131765386 | \_ |
| pos\_1972 | Asparaginyl-Glutamine | HMDB0028729 | Organic compounds | Organic acids and derivatives | Carboxylic acids and derivatives | Amino acids, peptides, and analogues | 446922 | 14608-83-4 |
| pos\_1979 | DG(20:2n6/0:0/18:3n3) | HMDB0056315 | Organic compounds | Lipids and lipid-like molecules | Fatty Acyls | Eicosanoids | 131802014 | \_ |
| pos\_1986 | Dihydrobiopterin | HMDB0000038 | Organic compounds | Organoheterocyclic compounds | Pteridines and derivatives | Pterins and derivatives | 119055 | 6779-87-9 |
| pos\_199 | alpha-curcumene | HMDB0061838 | Organic compounds | Lipids and lipid-like molecules | Prenol lipids | Sesquiterpenoids | 442360 | 4176-17-4 |
| pos\_1990 | LysoPE(0:0/20:1(11Z)) | HMDB0011482 | Organic compounds | Lipids and lipid-like molecules | Glycerophospholipids | Glycerophosphoethanolamines | 53480931 | \_ |
| pos\_1993 | PE(22:4(7Z,10Z,13Z,16Z)/24:0) | HMDB0009606 | Organic compounds | Lipids and lipid-like molecules | Glycerophospholipids | Glycerophosphoethanolamines | 53479903 | \_ |
| pos\_1998 | DG(20:0/0:0/18:2n6) | HMDB0056073 | Organic compounds | Lipids and lipid-like molecules | Glycerolipids | Diradylglycerols | 131801787 | \_ |
| pos\_2 | Betaine | HMDB0000043 | Organic compounds | Organic acids and derivatives | Carboxylic acids and derivatives | Amino acids, peptides, and analogues | 248 | 6915-17-9 |
| pos\_2000 | TG(22:1(13Z)/18:4(6Z,9Z,12Z,15Z)/22:6(4Z,7Z,10Z,13Z,16Z,19Z)) | HMDB0051831 | Organic compounds | Lipids and lipid-like molecules | Glycerolipids | Triradylcglycerols | 131762529 | \_ |
| pos\_2004 | N-Alpha-acetyllysine | HMDB0000446 | Organic compounds | Organic acids and derivatives | Carboxylic acids and derivatives | Amino acids, peptides, and analogues | 92907 | 1946-82-3 |
| pos\_2010 | Epilubimin | HMDB0035032 | Organic compounds | Lipids and lipid-like molecules | Prenol lipids | Sesquiterpenoids | 3504626 | 64024-09-5 |
| pos\_2012 | Asparaginyl-Cysteine | HMDB0028728 | Organic compounds | Organic acids and derivatives | Carboxylic acids and derivatives | Amino acids, peptides, and analogues | 54338059 | 71190-89-1 |
| pos\_2013 | Kelampayoside A | HMDB0038714 | Organic compounds | Organic oxygen compounds | Organooxygen compounds | Carbohydrates and carbohydrate conjugates | 10552637 | 87562-76-3 |
| pos\_2019 | Chinenoside III | HMDB0041343 | Organic compounds | Lipids and lipid-like molecules | Steroids and steroid derivatives | Steroidal glycosides | 131753113 | 172519-52-7 |
| pos\_2028 | Dihydrozeatin | HMDB0012215 | Organic compounds | Organoheterocyclic compounds | Imidazopyrimidines | Purines and purine derivatives | 439631 | 14742-97-3 |
| pos\_203 | Malvidin | HMDB0131420 | Organic compounds | Phenylpropanoids and polyketides | Flavonoids | O-methylated flavonoids | 69512 | \_ |
| pos\_2035 | PE(14:1(9Z)/22:2(13Z,16Z)) | HMDB0008876 | Organic compounds | Lipids and lipid-like molecules | Glycerophospholipids | Glycerophosphoethanolamines | 52924154 | \_ |
| pos\_2048 | Phenylacetaldehyde | HMDB0041610 | Organic compounds | Benzenoids | Benzene and substituted derivatives | \_ | 80584 | 6314-97-2 |
| pos\_2054 | Desmosterol | HMDB0002719 | Organic compounds | Lipids and lipid-like molecules | Steroids and steroid derivatives | Cholestane steroids | 439577 | 313-04-2 |
| pos\_2067 | PGP(18:0/20:3(5Z,8Z,11Z)) | HMDB0013510 | Organic compounds | Lipids and lipid-like molecules | Glycerophospholipids | Glycerophosphoglycerophosphates | 53481828 | \_ |
| pos\_2068 | DG(14:0/0:0/20:2n6) | HMDB0055969 | Organic compounds | Lipids and lipid-like molecules | Fatty Acyls | Eicosanoids | 131801701 | \_ |
| pos\_2085 | Asitrilobin D | HMDB0035389 | Organic compounds | Lipids and lipid-like molecules | Fatty Acyls | Fatty alcohols | 131751736 | \_ |
| pos\_2087 | DIBOA-Glc | HMDB0033734 | Organic compounds | Organic oxygen compounds | Organooxygen compounds | Carbohydrates and carbohydrate conjugates | 58114415 | 155835-54-4 |
| pos\_2096 | Austdiol | HMDB0030858 | Organic compounds | Organoheterocyclic compounds | Azaphilones | \_ | 3520232 | 53043-28-0 |
| pos\_21 | Acetoin | HMDB0003243 | Organic compounds | Organic oxygen compounds | Organooxygen compounds | Carbonyl compounds | 179 | 513-86-0 |
| pos\_2108 | PC(20:4(8Z,11Z,14Z,17Z)/22:2(13Z,16Z)) | HMDB0008481 | Organic compounds | Lipids and lipid-like molecules | Glycerophospholipids | Glycerophosphocholines | 53479101 | \_ |
| pos\_2116 | Garcimangosone D | HMDB0038066 | Organic compounds | Organic oxygen compounds | Organooxygen compounds | Carbohydrates and carbohydrate conjugates | 85362951 | \_ |
| pos\_2118 | Serinyl-Tyrosine | HMDB0029051 | Organic compounds | Organic acids and derivatives | Carboxylic acids and derivatives | Amino acids, peptides, and analogues | 7021865 | 21435-27-8 |
| pos\_2119 | PS(14:0/14:0) | HMDB0012330 | Organic compounds | Lipids and lipid-like molecules | Glycerophospholipids | Glycerophosphoserines | 123699 | \_ |
| pos\_2127 | TG(22:4(7Z,10Z,13Z,16Z)/22:5(4Z,7Z,10Z,13Z,16Z)/22:6(4Z,7Z,10Z,13Z,16Z,19Z)) | HMDB0054845 | Organic compounds | Lipids and lipid-like molecules | Glycerolipids | Triradylcglycerols | 131765395 | \_ |
| pos\_2136 | Ornithokinin | HMDB0038224 | Organic compounds | Organic acids and derivatives | Carboxylic acids and derivatives | Amino acids, peptides, and analogues | 3082051 | 14042-92-3 |
| pos\_2138 | PS(16:0/18:1(9Z)) | HMDB0012357 | Organic compounds | Lipids and lipid-like molecules | Glycerophospholipids | Glycerophosphoserines | 5283499 | \_ |
| pos\_2142 | 3-carboxy-4-methyl-5-propyl-2-furanpropanoate | HMDB0061112 | Organic compounds | Lipids and lipid-like molecules | Fatty Acyls | Fatty acids and conjugates | 123979 | 86879-39-2 |
| pos\_2151 | PE(14:0/20:5(5Z,8Z,11Z,14Z,17Z)) | HMDB0008840 | Organic compounds | Lipids and lipid-like molecules | Glycerophospholipids | Glycerophosphoethanolamines | 52924938 | \_ |
| pos\_2154 | DG(14:0/0:0/18:2n6) | HMDB0055967 | Organic compounds | Lipids and lipid-like molecules | Glycerolipids | Diradylglycerols | 131801699 | \_ |
| pos\_2158 | PE(22:6(4Z,7Z,10Z,13Z,16Z,19Z)/24:1(15Z)) | HMDB0009707 | Organic compounds | Lipids and lipid-like molecules | Glycerophospholipids | Glycerophosphoethanolamines | 53479982 | \_ |
| pos\_2159 | PE(P-16:0/15:0) | HMDB0011337 | Organic compounds | Lipids and lipid-like molecules | Glycerophospholipids | Glycerophosphoethanolamines | 52925044 | \_ |
| pos\_2162 | 2,5-Dimethylpyrazine | HMDB0035248 | Organic compounds | Organoheterocyclic compounds | Diazines | Pyrazines | 7938 | 108-50-9 |
| pos\_2166 | Prostaglandin A2 | HMDB0002752 | Organic compounds | Lipids and lipid-like molecules | Fatty Acyls | Eicosanoids | 5280880 | 13345-50-1 |
| pos\_2169 | Sapiol | HMDB0033177 | Organic compounds | Lipids and lipid-like molecules | Fatty Acyls | Fatty alcohols | 185639 | 28484-70-0 |
| pos\_2182 | 5-Methylcytidine | HMDB0000982 | Organic compounds | Nucleosides, nucleotides, and analogues | Pyrimidine nucleosides | \_ | 92918 | 2140-61-6 |
| pos\_2195 | PG(18:0/18:0) | HMDB0010602 | Organic compounds | Lipids and lipid-like molecules | Glycerophospholipids | Glycerophosphoglycerols | 24779552 | \_ |
| pos\_22 | 6-pentadecyl Salicylic Acid | HMDB0031059 | Organic compounds | Benzenoids | Benzene and substituted derivatives | Benzoic acids and derivatives | 5388781 | 103904-73-0 |
| pos\_220 | Retinaldehyde | HMDB0002152 | Organic compounds | Lipids and lipid-like molecules | Prenol lipids | Retinoids | 5280490 | 564-87-4 |
| pos\_2204 | Diethanolamine | HMDB0004437 | Organic compounds | Organic nitrogen compounds | Organonitrogen compounds | Amines | 8113 | 111-42-2 |
| pos\_2205 | N-Methylnicotinium | HMDB0001009 | Organic compounds | Organoheterocyclic compounds | Pyridines and derivatives | Pyrrolidinylpyridines | 430 | 24554-05-0 |
| pos\_2206 | PE(18:4(6Z,9Z,12Z,15Z)/24:1(15Z)) | HMDB0009212 | Organic compounds | Lipids and lipid-like molecules | Glycerophospholipids | Glycerophosphoethanolamines | 53479704 | \_ |
| pos\_2218 | Bilirubin diglucuronide | HMDB0003325 | Organic compounds | Organoheterocyclic compounds | Tetrapyrroles and derivatives | Bilirubins | 5280817 | 17459-92-6 |
| pos\_2219 | LysoPE(0:0/22:5(4Z,7Z,10Z,13Z,16Z)) | HMDB0011494 | Organic compounds | Lipids and lipid-like molecules | Glycerophospholipids | Glycerophosphoethanolamines | 53480943 | \_ |
| pos\_2224 | DG(18:0/22:5(7Z,10Z,13Z,16Z,19Z)/0:0) | HMDB0007178 | Organic compounds | Lipids and lipid-like molecules | Glycerolipids | Diradylglycerols | 9543870 | \_ |
| pos\_2229 | PIP(16:0/22:4(10Z,13Z,16Z,19Z)) | HMDB0009922 | Organic compounds | Lipids and lipid-like molecules | Glycerophospholipids | Glycerophosphoinositol phosphates | 131750480 | \_ |
| pos\_2233 | L-gamma-glutamyl-L-leucine | HMDB0011171 | Organic compounds | Organic acids and derivatives | Carboxylic acids and derivatives | Amino acids, peptides, and analogues | 151023 | 2566-39-4 |
| pos\_2236 | DG(18:0/0:0/20:4n6) | HMDB0056052 | Organic compounds | Lipids and lipid-like molecules | Fatty Acyls | Eicosanoids | 117992833 | \_ |
| pos\_2258 | Isopentenyladenine-9-N-glucoside | HMDB0012240 | Organic compounds | Organic oxygen compounds | Organooxygen compounds | Carbohydrates and carbohydrate conjugates | 11675006 | 83087-94-9 |
| pos\_2262 | TG(24:0/24:0/22:5(4Z,7Z,10Z,13Z,16Z)) | HMDB0047165 | Organic compounds | Lipids and lipid-like molecules | Glycerolipids | Triradylcglycerols | 131758008 | \_ |
| pos\_2263 | LysoPC(20:2(11Z,14Z)) | HMDB0010392 | Organic compounds | Lipids and lipid-like molecules | Glycerophospholipids | Glycerophosphocholines | 52924053 | \_ |
| pos\_2268 | 12b-Hydroxy-5b-cholanoic acid | HMDB0002431 | Organic compounds | Lipids and lipid-like molecules | Steroids and steroid derivatives | Bile acids, alcohols and derivatives | 5283812 | 15173-23-6 |
| pos\_2274 | N-Acetylornithine | HMDB0240589 | Organic compounds | Organic acids and derivatives | Carboxylic acids and derivatives | Amino acids, peptides, and analogues | 193343 | 2185-16-2 |
| pos\_2277 | TG(15:0/18:4(6Z,9Z,12Z,15Z)/o-18:0) | HMDB0043684 | Organic compounds | Lipids and lipid-like molecules | Fatty Acyls | Lineolic acids and derivatives | 131754804 | \_ |
| pos\_2279 | Hydrocinnamic acid | HMDB0000764 | Organic compounds | Phenylpropanoids and polyketides | Phenylpropanoic acids | \_ | 107 | 501-52-0 |
| pos\_2288 | PE(24:1(15Z)/20:5(5Z,8Z,11Z,14Z,17Z)) | HMDB0009764 | Organic compounds | Lipids and lipid-like molecules | Glycerophospholipids | Glycerophosphoethanolamines | 53480036 | \_ |
| pos\_2292 | DG(18:2(9Z,12Z)/22:5(4Z,7Z,10Z,13Z,16Z)/0:0) | HMDB0007264 | Organic compounds | Lipids and lipid-like molecules | Glycerolipids | Diradylglycerols | 53478109 | \_ |
| pos\_2305 | PE(20:0/22:6(4Z,7Z,10Z,13Z,16Z,19Z)) | HMDB0009243 | Organic compounds | Lipids and lipid-like molecules | Glycerophospholipids | Glycerophosphoethanolamines | 52924877 | \_ |
| pos\_2308 | Bassic acid | HMDB0034526 | Organic compounds | Lipids and lipid-like molecules | Steroids and steroid derivatives | Hydroxysteroids | 3698770 | 465-01-0 |
| pos\_2310 | Arginyl-Hydroxyproline | HMDB0028710 | Organic compounds | Organic acids and derivatives | Carboxylic acids and derivatives | Amino acids, peptides, and analogues | 69045664 | 182002-02-4 |
| pos\_2314 | Capsiate | HMDB0034781 | Organic compounds | Benzenoids | Phenols | Methoxyphenols | 9873754 | \_ |
| pos\_2316 | 1'-O-Acetylpaxilline | HMDB0037530 | Organic compounds | Organoheterocyclic compounds | Naphthopyrans | \_ | 3081700 | 121998-08-1 |
| pos\_2321 | Rebaudioside A | HMDB0034950 | Organic compounds | Lipids and lipid-like molecules | Prenol lipids | Terpene glycosides | 6918840 | 58543-16-1 |
| pos\_2322 | Sumiki's acid | HMDB0002432 | Organic compounds | Organoheterocyclic compounds | Furans | Furoic acid and derivatives | 80642 | 6338-41-6 |
| pos\_2323 | Polymyxin B Sulfate | HMDB0014919 | Organic compounds | Organic Polymers | Polypeptides | \_ | 5702105 | 1405-20-5 |
| pos\_2338 | 10,13-epoxy-11,12-dimethyloctadeca-10,12-dienoic acid | HMDB0061645 | Organic compounds | Lipids and lipid-like molecules | Fatty Acyls | Fatty acids and conjugates | 13963865 | 57818-40-3 |
| pos\_2341 | PE(15:0/16:1(9Z)) | HMDB0008891 | Organic compounds | Lipids and lipid-like molecules | Glycerophospholipids | Glycerophosphoethanolamines | 52924932 | \_ |
| pos\_2351 | DG(18:1(11Z)/24:0/0:0) | HMDB0007209 | Organic compounds | Lipids and lipid-like molecules | Glycerolipids | Diradylglycerols | 53478084 | \_ |
| pos\_2356 | DG(16:1(9Z)/22:4(7Z,10Z,13Z,16Z)/0:0) | HMDB0007147 | Organic compounds | Lipids and lipid-like molecules | Glycerolipids | Diradylglycerols | 9543814 | \_ |
| pos\_2363 | Lacto-N-biose I | HMDB0006575 | Organic compounds | Organic oxygen compounds | Organooxygen compounds | Carbohydrates and carbohydrate conjugates | 5288905 | 50787-09-2 |
| pos\_2364 | Allixin | HMDB0040705 | Organic compounds | Organoheterocyclic compounds | Pyrans | Pyranones and derivatives | 86374 | 125263-70-9 |
| pos\_2369 | DG(22:1(13Z)/22:5(7Z,10Z,13Z,16Z,19Z)/0:0) | HMDB0007642 | Organic compounds | Lipids and lipid-like molecules | Glycerolipids | Diradylglycerols | 9543958 | \_ |
| pos\_2372 | Pelargonic acid | HMDB0000847 | Organic compounds | Lipids and lipid-like molecules | Fatty Acyls | Fatty acids and conjugates | 8158 | 112-05-0 |
| pos\_2379 | 3-Methylcyclohexanol | HMDB0031538 | Organic compounds | Organic oxygen compounds | Organooxygen compounds | Alcohols and polyols | 11566 | 591-23-1 |
| pos\_2382 | MG(18:0e/0:0/0:0) | HMDB0011143 | Organic compounds | Lipids and lipid-like molecules | Glycerolipids | Monoradylglycerols | 10860876 | \_ |
| pos\_2385 | Artobiloxanthone | HMDB0034021 | Organic compounds | Benzenoids | Benzene and substituted derivatives | Phenylpropanes | 12570 | 121748-25-2 |
| pos\_2389 | Aframodial | HMDB0036806 | Organic compounds | Lipids and lipid-like molecules | Prenol lipids | Diterpenoids | 131752056 | 71641-23-1 |
| pos\_2409 | CDP-glucose | HMDB0003369 | Organic compounds | Nucleosides, nucleotides, and analogues | Pyrimidine nucleotides | Pyrimidine nucleotide sugars | 439244 | 2906-23-2 |
| pos\_2419 | TG(10:0/10:0/8:0) | HMDB0071580 | Organic compounds | Lipids and lipid-like molecules | Glycerolipids | Triradylcglycerols | 73416180 | \_ |
| pos\_2425 | DG(18:0/18:3(9Z,12Z,15Z)/0:0) | HMDB0007163 | Organic compounds | Lipids and lipid-like molecules | Fatty Acyls | Lineolic acids and derivatives | 9543723 | \_ |
| pos\_2432 | LysoPE(0:0/24:1(15Z)) | HMDB0011498 | Organic compounds | Lipids and lipid-like molecules | Glycerophospholipids | Glycerophosphoethanolamines | 53480947 | \_ |
| pos\_2438 | Acetyl tributyl citrate | HMDB0034159 | Organic compounds | Organic acids and derivatives | Carboxylic acids and derivatives | Tetracarboxylic acids and derivatives | 6505 | 77-90-7 |
| pos\_2445 | Muricoreacin | HMDB0032086 | Organic compounds | Lipids and lipid-like molecules | Fatty Acyls | Fatty alcohols | 10258454 | \_ |
| pos\_2447 | Glutaminyl-Proline | HMDB0028805 | Organic compounds | Organic acids and derivatives | Carboxylic acids and derivatives | Amino acids, peptides, and analogues | 11736661 | 139370-58-4 |
| pos\_2458 | LysoPE(0:0/24:6(6Z,9Z,12Z,15Z,18Z,21Z)) | HMDB0011499 | Organic compounds | Lipids and lipid-like molecules | Glycerophospholipids | Glycerophosphoethanolamines | 53480948 | \_ |
| pos\_2459 | DG(22:4(7Z,10Z,13Z,16Z)/22:6(4Z,7Z,10Z,13Z,16Z,19Z)/0:0) | HMDB0007701 | Organic compounds | Lipids and lipid-like molecules | Glycerolipids | Diradylglycerols | 9543969 | \_ |
| pos\_2466 | Delta-Tocopherol | HMDB0002902 | Organic compounds | Lipids and lipid-like molecules | Prenol lipids | Quinone and hydroquinone lipids | 92094 | 119-13-1 |
| pos\_2474 | 1,3-Butadiene | HMDB0041792 | Organic compounds | Hydrocarbons | Unsaturated hydrocarbons | Olefins | 7845 | 106-99-0 |
| pos\_2480 | DG(18:2n6/0:0/22:4n6) | HMDB0056288 | Organic compounds | Lipids and lipid-like molecules | Glycerolipids | Diradylglycerols | 131801988 | \_ |
| pos\_2481 | 5alpha-Androst-16-en-3-one | HMDB0034406 | Organic compounds | Lipids and lipid-like molecules | Steroids and steroid derivatives | Androstane steroids | 6852393 | 18339-16-7 |
| pos\_2484 | TG(18:3(6Z,9Z,12Z)/20:5(5Z,8Z,11Z,14Z,17Z)/22:5(4Z,7Z,10Z,13Z,16Z)) | HMDB0053215 | Organic compounds | Lipids and lipid-like molecules | Glycerolipids | Triradylcglycerols | 131763865 | \_ |
| pos\_2485 | LysoPE(0:0/22:4(7Z,10Z,13Z,16Z)) | HMDB0011493 | Organic compounds | Lipids and lipid-like molecules | Glycerophospholipids | Glycerophosphoethanolamines | 53480942 | \_ |
| pos\_2487 | Orciprenaline-3-O-sulfate | HMDB0060609 | Organic compounds | Organic acids and derivatives | Organic sulfuric acids and derivatives | Arylsulfates | 145888 | \_ |
| pos\_2491 | 6-Hydroxyhexanoic acid | HMDB0012843 | Organic compounds | Organic acids and derivatives | Hydroxy acids and derivatives | Medium-chain hydroxy acids and derivatives | 14490 | 1191-25-9 |
| pos\_2493 | Aflatoxin B2 | HMDB0035208 | Organic compounds | Phenylpropanoids and polyketides | Coumarins and derivatives | Furanocoumarins | 23648 | 7220-81-7 |
| pos\_250 | DG(18:3(9Z,12Z,15Z)/18:3(9Z,12Z,15Z)/0:0) | HMDB0007308 | Organic compounds | Lipids and lipid-like molecules | Fatty Acyls | Lineolic acids and derivatives | 9543745 | \_ |
| pos\_2506 | Kentsin | HMDB0005776 | Organic compounds | Organic acids and derivatives | Carboxylic acids and derivatives | Amino acids, peptides, and analogues | 171541 | 56767-30-7 |
| pos\_2507 | PC(20:4(5Z,8Z,11Z,14Z)/22:1(13Z)) | HMDB0008447 | Organic compounds | Lipids and lipid-like molecules | Glycerophospholipids | Glycerophosphocholines | 53479039 | \_ |
| pos\_251 | Melanostatin | HMDB0005764 | Organic compounds | Organic acids and derivatives | Carboxylic acids and derivatives | Amino acids, peptides, and analogues | 92910 | 9083-38-9 |
| pos\_2514 | PI(18:1(11Z)/18:1(11Z)) | HMDB0009824 | Organic compounds | Lipids and lipid-like molecules | Glycerophospholipids | Glycerophosphoinositols | \_ | \_ |
| pos\_2531 | Cellulose, microcrystalline | HMDB0032197 | Organic compounds | Organic oxygen compounds | Organooxygen compounds | Carbohydrates and carbohydrate conjugates | 14055602 | 9004-34-6 |
| pos\_2536 | Dexfenfluramine | HMDB0015322 | Organic compounds | Benzenoids | Benzene and substituted derivatives | Phenethylamines | 66265 | 3239-44-9 |
| pos\_2538 | SM(d18:1/24:0) | HMDB0011697 | Organic compounds | Lipids and lipid-like molecules | Sphingolipids | Phosphosphingolipids | 44260127 | 60037-60-7 |
| pos\_2539 | Mangostenol | HMDB0039912 | Organic compounds | Organoheterocyclic compounds | Benzopyrans | 1-benzopyrans | 5495927 | 437711-43-8 |
| pos\_2541 | Histidinal | HMDB0012234 | Organic compounds | Organic nitrogen compounds | Organonitrogen compounds | Amines | 152657 | 23784-15-8 |
| pos\_2545 | Pipermethystine | HMDB0033486 | Organic compounds | Organoheterocyclic compounds | Pyridines and derivatives | Hydropyridines | 194391 | 71627-22-0 |
| pos\_2557 | TG(8:0/10:0/i-16:0) | HMDB0071364 | Organic compounds | Lipids and lipid-like molecules | Glycerolipids | Triradylcglycerols | 131778170 | \_ |
| pos\_2559 | PE(14:0/14:0) | HMDB0008821 | Organic compounds | Lipids and lipid-like molecules | Glycerophospholipids | Glycerophosphoethanolamines | 9852308 | 998-07-2 |
| pos\_2560 | DG(18:1(11Z)/22:5(7Z,10Z,13Z,16Z,19Z)/0:0) | HMDB0007207 | Organic compounds | Lipids and lipid-like molecules | Glycerolipids | Diradylglycerols | 53478082 | \_ |
| pos\_2561 | DG(20:2(11Z,14Z)/22:5(4Z,7Z,10Z,13Z,16Z)/0:0) | HMDB0007438 | Organic compounds | Lipids and lipid-like molecules | Glycerolipids | Diradylglycerols | 53478235 | \_ |
| pos\_2568 | Ramontoside | HMDB0038929 | Organic compounds | Lignans, neolignans and related compounds | Lignan glycosides | \_ | 14825498 | 133882-75-4 |
| pos\_2578 | TG(14:1(9Z)/14:1(9Z)/o-18:0) | HMDB0047907 | Organic compounds | Lipids and lipid-like molecules | Glycerolipids | Triradylcglycerols | 131758748 | \_ |
| pos\_2579 | DG(20:4(5Z,8Z,11Z,14Z)/14:0/0:0) | HMDB0007501 | Organic compounds | Lipids and lipid-like molecules | Glycerolipids | Diradylglycerols | 53478287 | \_ |
| pos\_2588 | Citroside A | HMDB0030370 | Organic compounds | Lipids and lipid-like molecules | Prenol lipids | Terpene glycosides | 14312560 | 120330-44-1 |
| pos\_2590 | Progoitrin | HMDB0034071 | Organic compounds | Organic oxygen compounds | Organooxygen compounds | Carbohydrates and carbohydrate conjugates | 12309644 | 585-95-5 |
| pos\_2599 | N-Acetylglutamic acid | HMDB0001138 | Organic compounds | Organic acids and derivatives | Carboxylic acids and derivatives | Amino acids, peptides, and analogues | 70914 | 1188-37-0 |
| pos\_260 | PA(18:3(6Z,9Z,12Z)/16:0) | HMDB0114976 | Organic compounds | Lipids and lipid-like molecules | Glycerophospholipids | Glycerophosphates | 52928963 | \_ |
| pos\_2601 | NAD | HMDB0001487 | Organic compounds | Nucleosides, nucleotides, and analogues | 5')-dinucleotides | \_ | 439153 | 58-68-4 |
| pos\_2612 | Thiosulfate | HMDB0060293 | Inorganic compounds | Homogeneous non-metal compounds | Non-metal oxoanionic compounds | Non-metal thiosulfates | 1085 | \_ |
| pos\_2613 | LPA(18:0e/0:0) | HMDB0011144 | Organic compounds | Lipids and lipid-like molecules | Glycerophospholipids | Glycerophosphates | 10432493 | \_ |
| pos\_2615 | PC(20:5(5Z,8Z,11Z,14Z,17Z)/20:1(11Z)) | HMDB0008505 | Organic compounds | Lipids and lipid-like molecules | Glycerophospholipids | Glycerophosphocholines | 52923367 | \_ |
| pos\_2624 | DL-2-Aminooctanoic acid | HMDB0000991 | Organic compounds | Organic acids and derivatives | Carboxylic acids and derivatives | Amino acids, peptides, and analogues | 69522 | 644-90-6 |
| pos\_2628 | Oleoylcarnitine | HMDB0005065 | Organic compounds | Lipids and lipid-like molecules | Fatty Acyls | Fatty acid esters | 46907933 | 38677-66-6 |
| pos\_2632 | PI(18:2(9Z,12Z)/22:2(13Z,16Z)) | HMDB0009856 | Organic compounds | Lipids and lipid-like molecules | Glycerophospholipids | Glycerophosphoinositols | \_ | \_ |
| pos\_2635 | Spirolide D | HMDB0030493 | Organic compounds | Organoheterocyclic compounds | Azepines | \_ | 10699960 | 170713-73-2 |
| pos\_2638 | Geranylfarnesyl diphosphate | HMDB0012231 | Organic compounds | Lipids and lipid-like molecules | Prenol lipids | Polyprenols | 5280659 | \_ |
| pos\_2639 | PI(16:0/18:0) | HMDB0009781 | Organic compounds | Lipids and lipid-like molecules | Glycerophospholipids | Glycerophosphoinositols | \_ | 182509-29-1 |
| pos\_2641 | PE(16:1(9Z)/22:0) | HMDB0008973 | Organic compounds | Lipids and lipid-like molecules | Glycerophospholipids | Glycerophosphoethanolamines | 52924238 | \_ |
| pos\_2649 | Chondroitin 4-sulfate | HMDB0000652 | Organic compounds | Organic oxygen compounds | Organooxygen compounds | Carbohydrates and carbohydrate conjugates | 70678540 | 24967-93-9 |
| pos\_2655 | PC(DiMe(9,3)/DiMe(13,5)) | HMDB0061419 | Organic compounds | Lipids and lipid-like molecules | Glycerophospholipids | Glycerophosphocholines | \_ | \_ |
| pos\_2656 | Scleroglucan | HMDB0029948 | Organic compounds | Organic oxygen compounds | Organooxygen compounds | Carbohydrates and carbohydrate conjugates | 131750928 | 39464-87-4 |
| pos\_2658 | Methylphosphate | HMDB0061711 | Organic compounds | Organic acids and derivatives | Organic phosphoric acids and derivatives | Phosphate esters | 13130 | 812-00-0 |
| pos\_2664 | DG(20:5(5Z,8Z,11Z,14Z,17Z)/22:2(13Z,16Z)/0:0) | HMDB0007581 | Organic compounds | Lipids and lipid-like molecules | Glycerolipids | Diradylglycerols | 9543932 | \_ |
| pos\_2670 | Rifaximin | HMDB0015351 | Organic compounds | Phenylpropanoids and polyketides | Macrolactams | \_ | 46783403 | 80621-81-4 |
| pos\_2679 | DG(15:0/18:1(11Z)/0:0) | HMDB0007072 | Organic compounds | Lipids and lipid-like molecules | Glycerolipids | Diradylglycerols | 53478004 | \_ |
| pos\_2682 | Prostaglandin E3 | HMDB0002664 | Organic compounds | Lipids and lipid-like molecules | Fatty Acyls | Eicosanoids | 5280937 | 802-31-3 |
| pos\_2683 | PS(18:3(9Z,12Z,15Z)/20:4(5Z,8Z,11Z,14Z)) | HMDB0012416 | Organic compounds | Lipids and lipid-like molecules | Glycerophospholipids | Glycerophosphoserines | 52925527 | \_ |
| pos\_2687 | 4-Fumarylacetoacetic acid | HMDB0062563 | Organic compounds | Organic acids and derivatives | Keto acids and derivatives | Medium-chain keto acids and derivatives | 122 | \_ |
| pos\_2695 | DG(15:0/16:1(9Z)/0:0) | HMDB0007070 | Organic compounds | Lipids and lipid-like molecules | Glycerolipids | Diradylglycerols | 53478003 | \_ |
| pos\_2703 | LysoPE(0:0/20:3(8Z,11Z,14Z)) | HMDB0011486 | Organic compounds | Lipids and lipid-like molecules | Glycerophospholipids | Glycerophosphoethanolamines | 53480935 | \_ |
| pos\_2710 | Glycodiazine | HMDB0015461 | Organic compounds | Benzenoids | Benzene and substituted derivatives | Benzenesulfonamides | 9565 | 339-44-6 |
| pos\_2716 | 3,5-Diiodothyronine | HMDB0000582 | Organic compounds | Organic acids and derivatives | Carboxylic acids and derivatives | Amino acids, peptides, and analogues | 123675 | 534-51-0 |
| pos\_2720 | Adenosyl cobyrinic acid a,c diamide | HMDB0060429 | Organic compounds | Organoheterocyclic compounds | Tetrapyrroles and derivatives | Corrinoids | \_ | \_ |
| pos\_2725 | Methyl succinate | HMDB0061879 | Organic compounds | Lipids and lipid-like molecules | Fatty Acyls | Fatty acid esters | 77487 | \_ |
| pos\_2729 | Myrcene | HMDB0038169 | Organic compounds | Lipids and lipid-like molecules | Prenol lipids | Monoterpenoids | 31253 | 123-35-3 |
| pos\_2730 | Histamine | HMDB0000870 | Organic compounds | Organic nitrogen compounds | Organonitrogen compounds | Amines | 774 | 51-45-6 |
| pos\_2731 | 2-Dehydro-D-gluconate | HMDB0011732 | Organic compounds | Organic oxygen compounds | Organooxygen compounds | Carbohydrates and carbohydrate conjugates | 50 | 91548-32-2 |
| pos\_2732 | Ethyl butanoate | HMDB0033889 | Organic compounds | Lipids and lipid-like molecules | Fatty Acyls | Fatty acid esters | 7762 | 105-54-4 |
| pos\_2743 | PG(16:0/16:1(9Z)) | HMDB0010571 | Organic compounds | Lipids and lipid-like molecules | Glycerophospholipids | Glycerophosphoglycerols | 52927174 | \_ |
| pos\_2744 | L-Palmitoylcarnitine | HMDB0000222 | Organic compounds | Lipids and lipid-like molecules | Fatty Acyls | Fatty acid esters | 11953816 | 2364-67-2 |
| pos\_2755 | PC(14:0/16:1(9Z)) | HMDB0007870 | Organic compounds | Lipids and lipid-like molecules | Glycerophospholipids | Glycerophosphocholines | 24778615 | \_ |
| pos\_2759 | L-Acetylcarnitine | HMDB0000201 | Organic compounds | Lipids and lipid-like molecules | Fatty Acyls | Fatty acid esters | 7045767 | 3040-38-8 |
| pos\_276 | Dihydrocoumarin | HMDB0036626 | Organic compounds | Phenylpropanoids and polyketides | 3,4-dihydrocoumarins | \_ | 660 | 119-84-6 |
| pos\_2760 | Myristoleic acid | HMDB0002000 | Organic compounds | Lipids and lipid-like molecules | Fatty Acyls | Fatty acids and conjugates | 5281119 | 544-64-9 |
| pos\_2769 | PC(16:1(9Z)/0:0) | HMDB0010383 | Organic compounds | Lipids and lipid-like molecules | Glycerophospholipids | Glycerophosphocholines | 24779461 | 76790-27-7 |
| pos\_277 | Costunolide | HMDB0036688 | Organic compounds | Lipids and lipid-like molecules | Prenol lipids | Terpene lactones | 5367559 | 553-21-9 |
| pos\_2785 | Raffinose | HMDB0003213 | Organic compounds | Organic oxygen compounds | Organooxygen compounds | Carbohydrates and carbohydrate conjugates | 439242 | 512-69-6 |
| pos\_2788 | 2-phenylethanol | HMDB0041615 | Organic compounds | Benzenoids | Benzene and substituted derivatives | \_ | 91978 | 56011-02-0 |
| pos\_2792 | 2'-Deoxycytidine | HMDB0000014 | Organic compounds | Nucleosides, nucleotides, and analogues | Pyrimidine nucleosides | Pyrimidine 2'-deoxyribonucleosides | 13711 | 951-77-9 |
| pos\_2794 | PE(18:1(11Z)/14:0) | HMDB0009019 | Organic compounds | Lipids and lipid-like molecules | Glycerophospholipids | Glycerophosphoethanolamines | 53479618 | \_ |
| pos\_2800 | DG(18:0/18:1(9Z)/0:0) | HMDB0007160 | Organic compounds | Lipids and lipid-like molecules | Glycerolipids | Diradylglycerols | 6443547 | \_ |
| pos\_2801 | PS(18:1(9Z)/0:0) | HMDB0240603 | Organic compounds | Lipids and lipid-like molecules | Glycerophospholipids | Glycerophosphoserines | 9547099 | 89319-60-8 |
| pos\_2802 | Ubiquinone Q1 | HMDB0002012 | Organic compounds | Lipids and lipid-like molecules | Prenol lipids | Quinone and hydroquinone lipids | 4462 | 727-81-1 |
| pos\_2813 | Histidinyl-Cysteine | HMDB0028882 | Organic compounds | Organic acids and derivatives | Carboxylic acids and derivatives | Amino acids, peptides, and analogues | 15294281 | 116833-93-3 |
| pos\_2816 | N6-Methyl-L-lysine | HMDB0002038 | Organic compounds | Organic acids and derivatives | Carboxylic acids and derivatives | Amino acids, peptides, and analogues | 164795 | 1188-07-4 |
| pos\_2819 | Diethyl decanedioate | HMDB0040429 | Organic compounds | Lipids and lipid-like molecules | Fatty Acyls | Fatty acid esters | 8049 | 110-40-7 |
| pos\_2825 | 1-Aminocyclohexanecarboxylic acid | HMDB0038249 | Organic compounds | Lipids and lipid-like molecules | Prenol lipids | Monoterpenoids | 89306 | 2756-56-1 |
| pos\_2826 | 4-Nonylphenol | HMDB0038982 | Organic compounds | Benzenoids | Phenols | 1-hydroxy-2-unsubstituted benzenoids | 1752 | 104-40-5 |
| pos\_2827 | Benzoylecgonine | HMDB0041836 | Organic compounds | Benzenoids | Benzene and substituted derivatives | Benzoic acids and derivatives | 442997 | 519-09-5 |
| pos\_2829 | Arginyl-Arginine | HMDB0028703 | Organic compounds | Organic acids and derivatives | Carboxylic acids and derivatives | Amino acids, peptides, and analogues | 151956 | 15483-27-9 |
| pos\_2830 | Zearalenone | HMDB0031752 | Organic compounds | Phenylpropanoids and polyketides | Macrolides and analogues | Zearalenones | 5375083 | 17924-92-4 |
| pos\_2832 | Ginsenoside Rg3 | HMDB0039546 | Organic compounds | Lipids and lipid-like molecules | Prenol lipids | Triterpenoids | 12901617 | 14197-60-5 |
| pos\_2842 | 13,14-dihydro-15-keto-PGF2alpha | HMDB0004685 | Organic compounds | Lipids and lipid-like molecules | Fatty Acyls | Eicosanoids | 5283039 | 27376-76-7 |
| pos\_2843 | d-Dethiobiotin | HMDB0003581 | Organic compounds | Lipids and lipid-like molecules | Fatty Acyls | Fatty acids and conjugates | 445027 | 533-48-2 |
| pos\_2844 | 6-Aminocaproic acid | HMDB0001901 | Organic compounds | Lipids and lipid-like molecules | Fatty Acyls | Fatty acids and conjugates | 564 | 60-32-2 |
| pos\_2850 | Sinapyl alcohol | HMDB0013070 | Organic compounds | Benzenoids | Phenols | Methoxyphenols | 5280507 | 537-33-7 |
| pos\_2853 | alpha-Tocotrienol | HMDB0006327 | Organic compounds | Lipids and lipid-like molecules | Prenol lipids | Quinone and hydroquinone lipids | 5282347 | 58864-81-6 |
| pos\_296 | Ichthyotherol | HMDB0031270 | Organic compounds | Hydrocarbons | Unsaturated hydrocarbons | Unsaturated aliphatic hydrocarbons | 31285 | 124-11-8 |
| pos\_298 | α-CEHC | HMDB0001518 | Organic compounds | Organoheterocyclic compounds | Benzopyrans | 1-benzopyrans | 9943542 | 4072-32-6 |
| pos\_3 | Cholic acid | HMDB0000619 | Organic compounds | Lipids and lipid-like molecules | Steroids and steroid derivatives | Bile acids, alcohols and derivatives | 221493 | 81-25-4 |
| pos\_300 | Stearoylethanolamide | HMDB0013078 | Organic compounds | Organic nitrogen compounds | Organonitrogen compounds | Amines | 27902 | 111-57-9 |
| pos\_301 | Cycloviolaxanthin | HMDB0038106 | Organic compounds | Lipids and lipid-like molecules | Prenol lipids | Tetraterpenoids | 131752303 | 136624-30-1 |
| pos\_325 | Dibutyl sulfide | HMDB0031660 | Organic compounds | Organosulfur compounds | Thioethers | Dialkylthioethers | 11002 | 544-40-1 |
| pos\_331 | PE(22:2(13Z,16Z)/18:4(6Z,9Z,12Z,15Z)) | HMDB0009558 | Organic compounds | Lipids and lipid-like molecules | Glycerophospholipids | Glycerophosphoethanolamines | 52924788 | \_ |
| pos\_36 | Phenol | HMDB0013674 | Organic compounds | Benzenoids | Phenols | Benzenetriols and derivatives | 1057 | 87-66-1 |
| pos\_363 | o-Cymene | HMDB0037050 | Organic compounds | Benzenoids | Benzene and substituted derivatives | Cumenes | 10703 | 527-84-4 |
| pos\_368 | Phthalic acid Mono-2-ethylhexyl Ester | HMDB0013248 | Organic compounds | Benzenoids | Benzene and substituted derivatives | Benzoic acids and derivatives | 20393 | 4376-20-9 |
| pos\_37 | 1,2-Didecanoyl-sn-glycerol | HMDB0092961 | Organic compounds | Lipids and lipid-like molecules | Glycerolipids | Diradylglycerols | 162916 | \_ |
| pos\_387 | 7-Ethoxy-4-methyl-2H-1-benzopyran-2-one | HMDB0035095 | Organic compounds | Phenylpropanoids and polyketides | Coumarins and derivatives | \_ | 66595 | 87-05-8 |
| pos\_41 | Mesitylene | HMDB0041924 | Organic compounds | Benzenoids | Benzene and substituted derivatives | \_ | 7947 | 108-67-8 |
| pos\_423 | PE(18:4(6Z,9Z,12Z,15Z)/22:6(4Z,7Z,10Z,13Z,16Z,19Z)) | HMDB0009210 | Organic compounds | Lipids and lipid-like molecules | Glycerophospholipids | Glycerophosphoethanolamines | 52924469 | \_ |
| pos\_431 | PPA | HMDB0041989 | Organic compounds | Organoheterocyclic compounds | Diazinanes | Piperazines | 4831 | 51940-44-4 |
| pos\_446 | Idoxanthin | HMDB0040656 | Organic compounds | Lipids and lipid-like molecules | Prenol lipids | Tetraterpenoids | 131752890 | 97169-13-6 |
| pos\_461 | Palmitic amide | HMDB0012273 | Organic compounds | Lipids and lipid-like molecules | Fatty Acyls | Fatty amides | 69421 | 629-54-9 |
| pos\_462 | Stearoylglycine | HMDB0013308 | Organic compounds | Organic acids and derivatives | Carboxylic acids and derivatives | Amino acids, peptides, and analogues | 95070 | \_ |
| pos\_467 | Capsanthin 3,6-epoxide | HMDB0033260 | Organic compounds | Lipids and lipid-like molecules | Prenol lipids | Tetraterpenoids | 131751400 | \_ |
| pos\_472 | Heliespirone A | HMDB0031972 | Organic compounds | Organic oxygen compounds | Organooxygen compounds | Carbonyl compounds | 75231864 | 202533-71-9 |
| pos\_477 | N-Glycolylneuraminic acid | HMDB0062502 | Organic compounds | Organic oxygen compounds | Organooxygen compounds | Carbohydrates and carbohydrate conjugates | 440001 | \_ |
| pos\_481 | Tetrahydro-2-furanmethanol | HMDB0031175 | Organic compounds | Organoheterocyclic compounds | Tetrahydrofurans | \_ | 7360 | 97-99-4 |
| pos\_492 | Phthalic acid | HMDB0002107 | Organic compounds | Benzenoids | Benzene and substituted derivatives | Benzoic acids and derivatives | 1017 | 88-99-3 |
| pos\_495 | Vildagliptin | HMDB0015596 | Organic compounds | Organic acids and derivatives | Carboxylic acids and derivatives | Amino acids, peptides, and analogues | 6918537 | 274901-16-5 |
| pos\_499 | Oleic acid | HMDB0062703 | Organic compounds | Lipids and lipid-like molecules | Fatty Acyls | Fatty acids and conjugates | 965 | 2027-47-6 |
| pos\_504 | (2E,4E)-2,4-Hexadienoic acid | HMDB0029581 | Organic compounds | Lipids and lipid-like molecules | Fatty Acyls | Fatty acids and conjugates | 643460 | 110-44-1 |
| pos\_514 | Alpha-Linolenoyl ethanolamide | HMDB0013624 | Organic compounds | Organic nitrogen compounds | Organonitrogen compounds | Amines | 5283449 | \_ |
| pos\_52 | Palmitoyl glucuronide | HMDB0010331 | Organic compounds | Lipids and lipid-like molecules | Fatty Acyls | Fatty acyl glycosides | 161223 | \_ |
| pos\_522 | Dihomo-linoleate (20:2n6) | HMDB0005060 | Organic compounds | Lipids and lipid-like molecules | Fatty Acyls | Fatty acids and conjugates | 6439848 | 5598-38-9 |
| pos\_527 | Gingerglycolipid C | HMDB0041095 | Organic compounds | Lipids and lipid-like molecules | Glycerolipids | Glycosylglycerols | 131753024 | 35949-86-1 |
| pos\_531 | 5-Methyldeoxycytidine | HMDB0002224 | Organic compounds | Nucleosides, nucleotides, and analogues | Pyrimidine nucleosides | Pyrimidine 2'-deoxyribonucleosides | 13279 | 838-07-3 |
| pos\_535 | Proline betaine | HMDB0004827 | Organic compounds | Organic acids and derivatives | Carboxylic acids and derivatives | Amino acids, peptides, and analogues | 115244 | 471-87-4 |
| pos\_536 | Glutamyl-Methionine | HMDB0028825 | Organic compounds | Organic acids and derivatives | Carboxylic acids and derivatives | Amino acids, peptides, and analogues | 6426949 | 4423-22-7 |
| pos\_537 | 2-(Methylthio)phenol | HMDB0041281 | Organic compounds | Organosulfur compounds | Thioethers | Aryl thioethers | 61261 | 1073-29-6 |
| pos\_540 | Nonadeca-10(Z)-enoic acid | HMDB0013622 | Organic compounds | Lipids and lipid-like molecules | Fatty Acyls | Fatty acids and conjugates | 5312513 | 73033-09-7 |
| pos\_542 | 1-(alpha-Methyl-4-(2-methylpropyl)benzeneacetate)-beta-D-Glucopyranuronic acid | HMDB0010343 | Organic compounds | Organic oxygen compounds | Organooxygen compounds | Carbohydrates and carbohydrate conjugates | 92278491 | 98649-76-4 |
| pos\_543 | Acetaminophen glucuronide | HMDB0010316 | Organic compounds | Organic oxygen compounds | Organooxygen compounds | Carbohydrates and carbohydrate conjugates | 83944 | 16110-10-4 |
| pos\_545 | NADPH | HMDB0000221 | Organic compounds | Nucleosides, nucleotides, and analogues | 5')-dinucleotides | \_ | 5884 | 53-57-6 |
| pos\_549 | PG(18:0/22:6(4Z,7Z,10Z,13Z,16Z,19Z)) | HMDB0010614 | Organic compounds | Lipids and lipid-like molecules | Glycerophospholipids | Glycerophosphoglycerols | 24779554 | \_ |
| pos\_554 | Hypogeic acid | HMDB0002186 | Organic compounds | Lipids and lipid-like molecules | Fatty Acyls | Fatty acids and conjugates | 5318393 | 2416-19-5 |
| pos\_556 | 5-Hydroxylysine | HMDB0000450 | Organic compounds | Organic acids and derivatives | Carboxylic acids and derivatives | Amino acids, peptides, and analogues | 3032849 | 1190-94-9 |
| pos\_560 | Dodecanedioic acid | HMDB0000933 | Organic compounds | Lipids and lipid-like molecules | Fatty Acyls | Fatty acids and conjugates | 5283028 | 6402-36-4 |
| pos\_562 | 11beta-Hydroxy-3,20-dioxopregn-4-en-21-oic acid | HMDB0011652 | Organic compounds | Lipids and lipid-like molecules | Steroids and steroid derivatives | Androstane steroids | 53481012 | \_ |
| pos\_564 | 5-Hydroxyisourate | HMDB0030097 | Organic compounds | Organoheterocyclic compounds | Imidazopyrimidines | Purines and purine derivatives | 101957720 | 151359-24-9 |
| pos\_569 | Ethyl 2-methylpropanoate | HMDB0031248 | Organic compounds | Organic acids and derivatives | Carboxylic acids and derivatives | Carboxylic acid derivatives | 7342 | 97-62-1 |
| pos\_57 | 7-dehydrocholesterol | HMDB0000876 | Organic compounds | Lipids and lipid-like molecules | Steroids and steroid derivatives | Vitamin D and derivatives | 5280795 | 67-97-0 |
| pos\_574 | Oxymesterone | HMDB0006027 | Organic compounds | Lipids and lipid-like molecules | Steroids and steroid derivatives | Androstane steroids | 72061 | 145-12-0 |
| pos\_577 | Prostaglandin J2 | HMDB0004238 | Organic compounds | Lipids and lipid-like molecules | Fatty Acyls | Eicosanoids | 5280885 | 87893-54-7 |
| pos\_586 | 12S-HHT | HMDB0012535 | Organic compounds | Lipids and lipid-like molecules | Fatty Acyls | Fatty acids and conjugates | 5283141 | 54397-84-1 |
| pos\_590 | Asteltoxin | HMDB0029464 | Organic compounds | Organoheterocyclic compounds | Furofurans | \_ | 131750872 | 79663-49-3 |
| pos\_591 | 19-Hydroxydeoxycorticosterone | HMDB0012612 | Organic compounds | Lipids and lipid-like molecules | Steroids and steroid derivatives | Hydroxysteroids | 53481497 | 2394-23-2 |
| pos\_592 | Frangulanine | HMDB0030199 | Organic compounds | Organic acids and derivatives | Carboxylic acids and derivatives | Amino acids, peptides, and analogues | 5317388 | 25350-22-5 |
| pos\_593 | Glucosamine 6-sulfate | HMDB0000592 | Organic compounds | Organic oxygen compounds | Organooxygen compounds | Carbohydrates and carbohydrate conjugates | 72361 | 91674-26-9 |
| pos\_594 | 19-Norandrosterone | HMDB0005886 | Organic compounds | Lipids and lipid-like molecules | Steroids and steroid derivatives | Estrane steroids | 14009228 | 33036-33-8 |
| pos\_597 | Heteroxanthin | HMDB0001991 | Organic compounds | Organoheterocyclic compounds | Imidazopyrimidines | Purines and purine derivatives | 68374 | 552-62-5 |
| pos\_598 | Boswellic acid | HMDB0002388 | Organic compounds | Lipids and lipid-like molecules | Prenol lipids | Triterpenoids | 168928 | 631-69-6 |
| pos\_602 | 2'-Deoxyinosine triphosphate | HMDB0003537 | Organic compounds | Nucleosides, nucleotides, and analogues | Purine nucleotides | Purine deoxyribonucleotides | 146302 | 16595-02-1 |
| pos\_604 | Isobutyryl-L-carnitine | HMDB0000736 | Organic compounds | Lipids and lipid-like molecules | Fatty Acyls | Fatty acid esters | 168379 | 25518-49-4 |
| pos\_606 | 6-Deoxocastasterone | HMDB0033984 | Organic compounds | Lipids and lipid-like molecules | Steroids and steroid derivatives | Bile acids, alcohols and derivatives | 13870433 | 87833-54-3 |
| pos\_61 | 5-Aminoimidazole | HMDB0003929 | Organic compounds | Organoheterocyclic compounds | Azoles | Imidazoles | 484 | 4919-03-3 |
| pos\_614 | Fumaric acid | HMDB0000134 | Organic compounds | Organic acids and derivatives | Carboxylic acids and derivatives | Dicarboxylic acids and derivatives | 444972 | 110-17-8 |
| pos\_615 | N-lactoyl-Leucine | HMDB0062176 | Organic compounds | Organic acids and derivatives | Carboxylic acids and derivatives | Amino acids, peptides, and analogues | 57329455 | 210769-82-7 |
| pos\_625 | Momilactone B | HMDB0036749 | Organic compounds | Organoheterocyclic compounds | Naphthopyrans | \_ | 73018629 | 51415-08-8 |
| pos\_627 | Cerebronic acid | HMDB0039540 | Organic compounds | Lipids and lipid-like molecules | Fatty Acyls | Fatty acids and conjugates | 102430 | 544-57-0 |
| pos\_633 | DG(18:1(11Z)/18:3(6Z,9Z,12Z)/0:0) | HMDB0007191 | Organic compounds | Lipids and lipid-like molecules | Fatty Acyls | Lineolic acids and derivatives | 53478066 | \_ |
| pos\_634 | Azythromycin | HMDB0014352 | Organic compounds | Organic oxygen compounds | Organooxygen compounds | Carbohydrates and carbohydrate conjugates | 447043 | 83905-01-5 |
| pos\_638 | 3-Methyl-2-butenal | HMDB0012157 | Organic compounds | Organic oxygen compounds | Organooxygen compounds | Carbonyl compounds | 61020 | 107-86-8 |
| pos\_648 | 2-Hexyl-3-phenyl-2-propenal | HMDB0031736 | Organic compounds | Phenylpropanoids and polyketides | Cinnamaldehydes | \_ | 1550884 | 101-86-0 |
| pos\_650 | 4-Hydroxyproline | HMDB0240251 | Organic compounds | Organic acids and derivatives | Carboxylic acids and derivatives | Amino acids, peptides, and analogues | 440015 | 618-27-9 |
| pos\_656 | O-Desmethylangolensin | HMDB0004629 | Organic compounds | Phenylpropanoids and polyketides | Alpha-methyldeoxybenzoin flavonoids | \_ | 89472 | 21255-69-6 |
| pos\_662 | All-trans-retinoic acid | HMDB0001852 | Organic compounds | Lipids and lipid-like molecules | Prenol lipids | Retinoids | 444795 | 302-79-4 |
| pos\_664 | DG(15:0/20:1(11Z)/0:0) | HMDB0007079 | Organic compounds | Lipids and lipid-like molecules | Glycerolipids | Diradylglycerols | 53478008 | \_ |
| pos\_668 | N-Methyl-1-deoxynojirimycin | HMDB0035360 | Organic compounds | Organoheterocyclic compounds | Piperidines | \_ | 4381 | 69567-10-8 |
| pos\_671 | L-Methionine | HMDB0033951 | Organic compounds | Organic acids and derivatives | Carboxylic acids and derivatives | Amino acids, peptides, and analogues | 876 | 59-51-8 |
| pos\_674 | 12-Hydroxydodecanoic acid | HMDB0002059 | Organic compounds | Organic acids and derivatives | Hydroxy acids and derivatives | Medium-chain hydroxy acids and derivatives | 79034 | 505-95-3 |
| pos\_676 | Oxamniquine | HMDB0015228 | Organic compounds | Organoheterocyclic compounds | Quinolines and derivatives | Nitroquinolines and derivatives | 4612 | 21738-42-1 |
| pos\_683 | Linamarin | HMDB0005008 | Organic compounds | Organoheterocyclic compounds | Benzimidazoles | Sulfinylbenzimidazoles | 3883 | 103577-45-3 |
| pos\_687 | Ganoderic acid A | HMDB0034931 | Organic compounds | Lipids and lipid-like molecules | Prenol lipids | Triterpenoids | 14109402 | 81907-62-2 |
| pos\_69 | 3-hydroxyadipic acid 3,6-lactone | HMDB0029171 | Organic compounds | Organoheterocyclic compounds | Lactones | Gamma butyrolactones | 194109 | 60551-20-4 |
| pos\_691 | Hydroxyisonobilin | HMDB0034475 | Organic compounds | Lipids and lipid-like molecules | Prenol lipids | Terpene lactones | 131751567 | \_ |
| pos\_694 | Glucosylsphingosine | HMDB0000596 | Organic compounds | Lipids and lipid-like molecules | Sphingolipids | Glycosphingolipids | 22833534 | 52050-17-6 |
| pos\_695 | Gamma-Linolenic acid | HMDB0003073 | Organic compounds | Lipids and lipid-like molecules | Fatty Acyls | Lineolic acids and derivatives | 5280933 | 506-26-3 |
| pos\_701 | DG(18:1(11Z)/22:6(4Z,7Z,10Z,13Z,16Z,19Z)/0:0) | HMDB0007208 | Organic compounds | Lipids and lipid-like molecules | Glycerolipids | Diradylglycerols | 53478083 | \_ |
| pos\_702 | CE(18:3(6Z,9Z,12Z)) | HMDB0010369 | Organic compounds | Lipids and lipid-like molecules | Steroids and steroid derivatives | Steroid esters | 53480458 | \_ |
| pos\_705 | 1-(8Z,11Z,14Z-eicosatrienoyl)-glycero-3-phosphate | HMDB0114759 | Organic compounds | Lipids and lipid-like molecules | Glycerophospholipids | Glycerophosphates | 87978475 | \_ |
| pos\_709 | 5-Aminopentanoic acid | HMDB0003355 | Organic compounds | Organic acids and derivatives | Carboxylic acids and derivatives | Amino acids, peptides, and analogues | 138 | 660-88-8 |
| pos\_717 | 2-Hydroxybutyric acid | HMDB0000008 | Organic compounds | Organic acids and derivatives | Hydroxy acids and derivatives | Alpha hydroxy acids and derivatives | 440864 | 3347-90-8 |
| pos\_718 | PC(14:0/18:4(6Z,9Z,12Z,15Z)) | HMDB0007877 | Organic compounds | Lipids and lipid-like molecules | Glycerophospholipids | Glycerophosphocholines | 24778627 | \_ |
| pos\_720 | Zedoarondiol | HMDB0036448 | Organic compounds | Lipids and lipid-like molecules | Prenol lipids | Sesquiterpenoids | 14632997 | 98644-24-7 |
| pos\_722 | Eicosenoic acid | HMDB0033022 | Organic compounds | Lipids and lipid-like molecules | Fatty Acyls | Fatty acids and conjugates | 5282768 | 135357-25-4 |
| pos\_730 | CE(22:6(4Z,7Z,10Z,13Z,16Z,19Z)) | HMDB0006733 | Organic compounds | Lipids and lipid-like molecules | Steroids and steroid derivatives | Steroid esters | 53477890 | 70110-50-8 |
| pos\_733 | Valyl-Serine | HMDB0029136 | Organic compounds | Organic acids and derivatives | Carboxylic acids and derivatives | Amino acids, peptides, and analogues | 6992639 | 13588-94-8 |
| pos\_743 | Crustecdysone | HMDB0030180 | Organic compounds | Lipids and lipid-like molecules | Steroids and steroid derivatives | Bile acids, alcohols and derivatives | 5459840 | 5289-74-7 |
| pos\_752 | 3-Methoxy-4-hydroxyphenylglycol glucuronide | HMDB0000496 | Organic compounds | Organic oxygen compounds | Organooxygen compounds | Carbohydrates and carbohydrate conjugates | 22833525 | 52002-41-2 |
| pos\_755 | Undecylenic acid | HMDB0033724 | Organic compounds | Lipids and lipid-like molecules | Fatty Acyls | Fatty acids and conjugates | 5634 | 112-38-9 |
| pos\_759 | 5-L-Glutamyl-L-alanine | HMDB0006248 | Organic compounds | Organic acids and derivatives | Carboxylic acids and derivatives | Amino acids, peptides, and analogues | 440103 | 5875-41-2 |
| pos\_761 | 3,4-Methylenedioxyamphetamine | HMDB0041931 | Organic compounds | Organoheterocyclic compounds | Benzodioxoles | \_ | 1614 | 4764-17-4 |
| pos\_763 | PC(o-16:0/20:4(8Z,11Z,14Z,17Z)) | HMDB0013407 | Organic compounds | Lipids and lipid-like molecules | Glycerophospholipids | Glycerophosphocholines | 53481701 | \_ |
| pos\_764 | Mitomycin | HMDB0014450 | Organic compounds | Organoheterocyclic compounds | Indoles and derivatives | Indolequinones | 5746 | 50-07-7 |
| pos\_766 | L-Arginine | HMDB0062762 | Organic compounds | Organic acids and derivatives | Carboxylic acids and derivatives | Amino acids, peptides, and analogues | 1549073 | \_ |
| pos\_767 | N(6)-Methyllysine | HMDB0002038 | Organic compounds | Organic acids and derivatives | Carboxylic acids and derivatives | Amino acids, peptides, and analogues | 164795 | 1188-07-4 |
| pos\_769 | Cucurbitaxanthin A | HMDB0035319 | Organic compounds | Lipids and lipid-like molecules | Prenol lipids | Tetraterpenoids | 131751704 | 103955-77-7 |
| pos\_77 | 1-Phenyl-1-pentanone | HMDB0031208 | Organic compounds | Organic oxygen compounds | Organooxygen compounds | Carbonyl compounds | 66093 | 1009-14-9 |
| pos\_770 | Valyl-Hydroxyproline | HMDB0029128 | Organic compounds | Organic acids and derivatives | Carboxylic acids and derivatives | Amino acids, peptides, and analogues | 10242944 | 90965-79-0 |
| pos\_779 | Neuroprotectin D1 | HMDB0003689 | Organic compounds | Lipids and lipid-like molecules | Fatty Acyls | Fatty acids and conjugates | 16042541 | \_ |
| pos\_78 | Chamazulene | HMDB0036470 | Organic compounds | Lipids and lipid-like molecules | Prenol lipids | Sesquiterpenoids | 10719 | 529-05-5 |
| pos\_782 | Dopamine 4-sulfate | HMDB0004148 | Organic compounds | Organic acids and derivatives | Organic sulfuric acids and derivatives | Arylsulfates | 123932 | 38339-02-5 |
| pos\_786 | Oleacein | HMDB0037321 | Organic compounds | Benzenoids | Phenols | Tyrosols and derivatives | 18684078 | 149183-75-5 |
| pos\_788 | Cucurbitacin D | HMDB0034695 | Organic compounds | Lipids and lipid-like molecules | Steroids and steroid derivatives | Cucurbitacins | 5353546 | 3877-86-9 |
| pos\_796 | DG(14:1(9Z)/22:2(13Z,16Z)/0:0) | HMDB0007059 | Organic compounds | Lipids and lipid-like molecules | Glycerolipids | Diradylglycerols | 53477994 | \_ |
| pos\_799 | Diacetone alcohol | HMDB0031511 | Organic compounds | Organic oxygen compounds | Organooxygen compounds | Carbonyl compounds | 31256 | 123-42-2 |
| pos\_800 | DG(18:0/20:4(8Z,11Z,14Z,17Z)/0:0) | HMDB0007171 | Organic compounds | Lipids and lipid-like molecules | Glycerolipids | Diradylglycerols | 53478053 | \_ |
| pos\_807 | Aldosine | HMDB0037817 | Organic compounds | Organic acids and derivatives | Carboxylic acids and derivatives | Amino acids, peptides, and analogues | 57357510 | 142759-11-3 |
| pos\_809 | Hexadecanedioic acid | HMDB0000712 | Organic compounds | Lipids and lipid-like molecules | Fatty Acyls | Fatty acid esters | 566787 | 42150-38-9 |
| pos\_813 | Phytosphingosine | HMDB0004610 | Organic compounds | Organic nitrogen compounds | Organonitrogen compounds | Amines | 122121 | 554-62-1 |
| pos\_814 | Hemorphin-4 | HMDB0059788 | Organic compounds | Organic acids and derivatives | Carboxylic acids and derivatives | Amino acids, peptides, and analogues | 128444 | \_ |
| pos\_816 | Imidazoleacetic acid ribotide | HMDB0006032 | Organic compounds | Organic oxygen compounds | Organooxygen compounds | Carbohydrates and carbohydrate conjugates | 16019958 | 2888-19-9 |
| pos\_819 | Endomorphin-2 | HMDB0005774 | Organic compounds | Organic acids and derivatives | Carboxylic acids and derivatives | Amino acids, peptides, and analogues | 5311081 | 141801-26-5 |
| pos\_825 | 1-(6Z,9Z,12Z-octadecatrienoyl)-glycero-3-phosphate | HMDB0114743 | Organic compounds | Lipids and lipid-like molecules | Glycerophospholipids | Glycerophosphates | 131821848 | \_ |
| pos\_827 | DG(14:1(9Z)/22:6(4Z,7Z,10Z,13Z,16Z,19Z)/0:0) | HMDB0007063 | Organic compounds | Lipids and lipid-like molecules | Glycerolipids | Diradylglycerols | 53477998 | \_ |
| pos\_829 | Blasticidin S | HMDB0030452 | Organic compounds | Organic acids and derivatives | Carboxylic acids and derivatives | Amino acids, peptides, and analogues | 258 | 2079-00-7 |
| pos\_835 | 3-Dehydroxycarnitine | HMDB0001161 | Organic compounds | Lipids and lipid-like molecules | Fatty Acyls | Fatty acids and conjugates | 725 | 407-64-7 |
| pos\_840 | DG(20:3(5Z,8Z,11Z)/22:6(4Z,7Z,10Z,13Z,16Z,19Z)/0:0) | HMDB0007469 | Organic compounds | Lipids and lipid-like molecules | Glycerolipids | Diradylglycerols | 53478264 | \_ |
| pos\_863 | trans-1,2-Dihydrobenzene-1,2-diol | HMDB0001164 | Organic compounds | Organic oxygen compounds | Organooxygen compounds | Alcohols and polyols | 149186 | 103302-38-1 |
| pos\_866 | N-Oleoylethanolamine | HMDB0002088 | Organic compounds | Organic nitrogen compounds | Organonitrogen compounds | Amines | 5283454 | 111-58-0 |
| pos\_878 | 7-Ketodeoxycholic acid | HMDB0000391 | Organic compounds | Lipids and lipid-like molecules | Steroids and steroid derivatives | Bile acids, alcohols and derivatives | 188292 | 911-40-0 |
| pos\_879 | Acetylcholine | HMDB0000895 | Organic compounds | Organic nitrogen compounds | Organonitrogen compounds | Quaternary ammonium salts | 187 | 51-84-3 |
| pos\_883 | Tsugarioside B | HMDB0035509 | Organic compounds | Lipids and lipid-like molecules | Prenol lipids | Triterpenoids | 85202365 | 267649-97-8 |
| pos\_884 | Anandamide | HMDB0004080 | Organic compounds | Organic nitrogen compounds | Organonitrogen compounds | Amines | 5281969 | 94421-68-8 |
| pos\_889 | PI(16:0/20:0) | HMDB0009785 | Organic compounds | Lipids and lipid-like molecules | Glycerophospholipids | Glycerophosphoinositols | \_ | \_ |
| pos\_891 | Hericenone D | HMDB0039139 | Organic compounds | Lipids and lipid-like molecules | Prenol lipids | Monoterpenoids | 15658906 | 137592-04-2 |
| pos\_9 | Retinoic acid | HMDB0006219 | Organic compounds | Lipids and lipid-like molecules | Prenol lipids | Retinoids | 5282379 | 4759-48-2 |
| pos\_916 | Lysyl-Proline | HMDB0028959 | Organic compounds | Organic acids and derivatives | Carboxylic acids and derivatives | Amino acids, peptides, and analogues | 171204 | 52766-27-5 |
| pos\_919 | 4-Hydroxyandrostenedione glucuronide | HMDB0010315 | Organic compounds | Lipids and lipid-like molecules | Steroids and steroid derivatives | Cholestane steroids | 443212 | \_ |
| pos\_926 | Isoleucyl-Hydroxyproline | HMDB0028908 | Organic compounds | Organic acids and derivatives | Carboxylic acids and derivatives | Amino acids, peptides, and analogues | 61158802 | 90965-80-3 |
| pos\_929 | Fagomine | HMDB0033453 | Organic compounds | Organoheterocyclic compounds | Piperidines | \_ | 72259 | 53185-12-9 |
| pos\_932 | Falcarindiol | HMDB0033941 | Organic compounds | Lipids and lipid-like molecules | Fatty Acyls | Fatty alcohols | 5317309 | 55297-87-5 |
| pos\_936 | Neohesperidin dihydrochalcone | HMDB0030542 | Organic compounds | Phenylpropanoids and polyketides | Flavonoids | Flavonoid glycosides | 4453 | 20702-77-6 |
| pos\_963 | Permetin A | HMDB0030527 | Organic compounds | Organic acids and derivatives | Peptidomimetics | Depsipeptides | 172928 | 71888-70-5 |
| pos\_970 | Hydroxydestruxin B | HMDB0040135 | Organic compounds | Organic acids and derivatives | Peptidomimetics | Depsipeptides | 85220065 | \_ |
| pos\_976 | Nonoxynol-9 | HMDB0015680 | Organic compounds | Organic oxygen compounds | Organooxygen compounds | Ethers | 72385 | 26571-11-9 |
| pos\_978 | LysoPE(0:0/20:2(11Z,14Z)) | HMDB0011483 | Organic compounds | Lipids and lipid-like molecules | Glycerophospholipids | Glycerophosphoethanolamines | 53480932 | \_ |
| pos\_980 | N-Carbamoylputrescine | HMDB0033458 | Organic compounds | Organic acids and derivatives | Carboximidic acids and derivatives | Carboximidic acids | 502 | 6851-51-0 |
| pos\_982 | Histidinyl-Lysine | HMDB0028890 | Organic compounds | Benzenoids | Phenol ethers | Anisoles | 148224 | 37700-85-9 |
| pos\_994 | Ponasteroside A | HMDB0034091 | Organic compounds | Lipids and lipid-like molecules | Steroids and steroid derivatives | Steroidal glycosides | 12314455 | 20117-33-3 |
| pos\_999 | Tryptophyl-Proline | HMDB0029091 | Organic compounds | Organic acids and derivatives | Carboxylic acids and derivatives | Amino acids, peptides, and analogues | 14409732 | \_ |
